# Visualization formats of patient-reported outcome measures in clinical practice: a systematic review about preferences and interpretation accuracy

**DOI:** 10.1186/s41687-022-00424-3

**Published:** 2022-03-03

**Authors:** Elaine A. C. Albers, Itske Fraterman, Iris Walraven, Erica Wilthagen, Sanne B. Schagen, Iris M. van der Ploeg, Michel W. J. M. Wouters, Lonneke V. van de Poll-Franse, Kelly M. de Ligt

**Affiliations:** 1 Department of Psychosocial Research, Division of Psychosocial Research and Epidemiology, The Netherlands Cancer Institute, Amsterdam, The Netherlands; 2 Department for Health Evidence, Radboudumc, Nijmegen, The Netherlands; 3 Library and Scientific Information Department, The Netherlands Cancer Institute, Amsterdam, The Netherlands; 4 Department of Psychology, University of Amsterdam, Amsterdam, The Netherlands; 5 Department of Surgical Oncology, Antoni Van Leeuwenhoek, Amsterdam, The Netherlands; 6 Department of Biomedical Data Sciences, Leiden University Medical Center, Leiden, The Netherlands; 7 Department of Research and Development, Netherlands Comprehensive Cancer Organization, Utrecht, The Netherlands; 8 Department of Medical and Clinical Psychology, Center of Research On Psychological and Somatic Disorders (CoRPS), Tilburg University, Tilburg, The Netherlands

**Keywords:** Patient reported outcome measures, Data visualization, Shared decision-making, Systematic review

## Abstract

**Purpose:**

The use of Patient-Reported Outcome Measures (PROMs) for individual patient management within clinical practice is becoming increasingly important. New evidence about graphic visualization formats for PROMs scores has become available. This systematic literature review evaluated evidence for graphic visualization formats of PROMs data in clinical practice for patients and clinicians, for both individual and group level PROMs data.

**Methods:**

Studies published between 2000 and 2020 were extracted from CINAHL, PubMed, PsychInfo, and Medline. Studies included patients ≥ 18 years old in daily clinical practice. Papers not available in English, without full-text access, or that did not specifically describe visualization of PROMs data were excluded. Outcomes were: visualization preferences; interpretation accuracy; guidance for clinical interpretation.

**Results:**

Twenty-five out of 789 papers were included for final analysis. Most frequently studied formats were: bar charts, line graphs, and pie charts. Patients preferred bar charts and line graphs as these were easy and quick for retrieving information about their PROMs scores over time. Clinicians’ interpretation accuracy and preferences were similar among graphic visualization formats. Scores were most often compared with patients’ own previous scores; to further guide clinical interpretation, scores were compared to norm population scores. Different ‘add-ons’ improved interpretability for patients and clinicians, e.g. using colors, descriptions of measurement scale directionality, descriptive labels, and brief definitions.

**Conclusion:**

There was no predominant graphical visualization format approach in terms of preferences or interpretation accuracy for both patients and clinicians. Detailed clarification of graph content is essential.

**Supplementary Information:**

The online version contains supplementary material available at 10.1186/s41687-022-00424-3.

## Plain English summary

Patient-Reported Outcome Measures (PROMs) capture patients' self-reported health through the use of questionnaires. PROMs measure health related quality of life, daily functioning, and symptom experience, which are becoming increasingly important to incorporate in clinical practice for individual patient management. To present PROMs within clinical practice, raw or summarized PROMs scores can be visualized in graphical formats. To be useful during clinical encounters, both patients and clinicians ought to interpret such formats correctly. New evidence about graphic visualization formats for PROMs scores has become available. Therefore, we systematically reviewed the literature to evaluate evidence for graphic visualization formats of PROMs data in clinical practice. In 25 included papers, most studies used graphical formats like bar charts, line graphs, and pie charts for presenting PROMs scores. There was no predominant graphical visualization format approach in terms of preferences or interpretation accuracy for both patients and clinicians. Patients preferred bar charts and line graphs as these were easy and quick for retrieving information about their PROMs scores over time. Clinicians’ interpretation accuracy and preferences were similar among graphic visualization formats. The graphical interpretation of PROMs data for patients and clinicians can be improved by using colors, descriptions of measurement scale directionality, descriptive labels, and brief definitions.

**Supplementary Information:**

The online version contains supplementary material available at 10.1186/s41687-022-00424-3.

## Introduction

With an increasing emphasis on patient-centred care, there is a growing interest in outcome measures most relevant to patients [1–6]. Patient-reported outcomes measures (PROMs) comprise data collected from individual patients and include an array of outcomes such as symptoms, daily functioning, and health-related quality of life (HRQoL). PROMs are increasingly used in daily clinical practice for individual patient management [7]. Individual PROMs data incorporates the patient’s perspective on their health status and can detect issues that are most bothersome to the individual patient. By reporting these issues to both patients and clinicians, patient-physician communication improves [8–10]. This may support shared-decision making, and therefore offers considerable potential to enhance quality of care and clinical outcomes [8, 11]. A second application of PROMs data feedback is the use of aggregated PROMs scores collected in clinical studies or trials to inform patients about treatment harms and benefits [12].

Currently, when PROMs are incorporated within clinical practice, raw or summarized PROMs data are given as feedback to patients and/or clinicians by using different graphic visualization formats [13]. In order for them to understand and apply the information during clinical encounters, patients and clinicians ought to interpret such formats correctly [13]. A previous review by Bantug et al. [13] reported that the majority of patients and clinicians were able to interpret plain or straightforward graphs. Bantug et al. suggested that future research should focus on optimizing graphic visualization strategies. After the publication of this review in 2016, considerable new evidence has become available about this topic. Moreover, the focus of recent studies has shifted towards the effect of aspects such as score directionality [14–17] and axis labelling [1, 15] on correct interpretation by patients and clinicians. Furthermore, there is increased attention for guiding the clinical interpretation of PROMs data, e.g. to distinguish severe or clinically relevant symptoms [1, 3–5, 14, 18]. For instance, the display of score thresholds and warnings if scores change over time would be helpful in daily practice [1], as well as scores from reference populations to compare individual scores to [1, 5, 14]. Both facilitate the correct use of scores during clinical encounters. The focus on clinical interpretation led to the introduction of funnel plots [2, 19], heat maps [4], and icon arrays [3, 15, 18, 20], underlining the relevance of assessing a wider variety of graphic visualization formats.

While worldwide implementation of PROMs data collection in clinical practice keeps progressing [21], new evidence on graphic visualization formats for PROMs scores for interpretation by patients and clinicians has become available. This systematic literature review aims to (1) address the latest evidence for graphic visualization formats of PROMs in clinical practice, by extracting preferences and interpretation accuracy for patient and clinicians, and (2) investigate how clinically relevant PROMs scores are distinguished, in order to guide clinical interpretation of PROMs scores for their use during clinical encounters.

## Methods

A systematic literature review was conducted according to the Preferred Reporting Items for Systematic Reviews and Meta-Analysis (PRISMA) guidelines [22].

### Search strategy

An exhaustive search strategy was developed by a medical librarian (E.W.) experienced in systematic literature searches. The following search terms were included: (“Patient reported outcome (PRO)” OR “Health-Related Quality of Life”) AND “data presentation/display” AND “health communication” AND (“cancer” OR “clinical decision-making”). We focused the search on oncology as we work in an oncological setting. Also, most of these studies have been conducted in oncology, as confirmed by our search and previously shown by Bantug et al. [13]. Literature from January 2000 to July 2020 was searched in MEDLINE (accessed through PubMed), Embase (assessed through Ovid Platform), PsycINFO (assessed through Ovid Platform), and CINAHL. The full search strategy is included in Additional file 1: Table 1. Duplicates were removed using the Bramer method [23]. A forward and backward reference check was performed on all final included articles.

### Review procedure

After removing duplicates, two researchers (E.A., I.F.) independently reviewed potential abstracts. The researchers eliminated articles according to the predefined inclusion and exclusion criteria (see below). In case of discrepancies, a third researcher (K.d.L.) was consulted. Subsequently, both researchers (E.A., I.F.) independently reviewed the full text version of each paper for inclusion in the final selection. Disagreements were discussed between the three researchers (E.A., I.F., and K.d.L.) to reach consensus.

Studies were included when concerning patients of 18 years and older treated in clinical practice; addressed communication of individual level or group level PROMs data, using graphic visualisation formats, to either patients or clinicians; were published in English. Exclusion criteria were: studies without English or full-text version; systematic literature reviews; Delphi studies; studies where PROMs had not been completed by patients or were not applied or visually presented to patients and/or clinicians.

### Analyses

Two researchers (E.A., K.d.L.) independently extracted data from the included articles. The findings of both researchers were compared and verified by a third researcher (I.F.). First, study characteristics were described, including study goal, population, and type of PROMs data that was visualised. Second, findings about visualisation preferences and interpretation accuracy were extracted. Furthermore, methods and strategies for guiding the interpretation of scores during clinical encounters were extracted. The findings were described for patients and clinicians separately, and a distinction was made between individual PROMs data for use during clinical encounters, and mean or aggregated PROMs data that could be included in treatment decision-making.

### Quality assessment

Two researchers (E.A., K.d.L.) independently assessed the methodological quality of the papers and compared their final judgments. The Critical Appraisal Skill Program (CASP) was used for methodological assessment of the included papers. CASP enables to systematically assess the trustworthiness, relevance, and results of published papers by comprising several criteria for qualitative studies, randomized controlled trials, and cohort studies [24]. The reviewers scored the papers per criteria with a positive or negative response, or ‘not applicable/unknown’. Studies with a positive score for half or more of the criteria were deemed of sufficient quality [24].

## Results

**Fig. 1:**
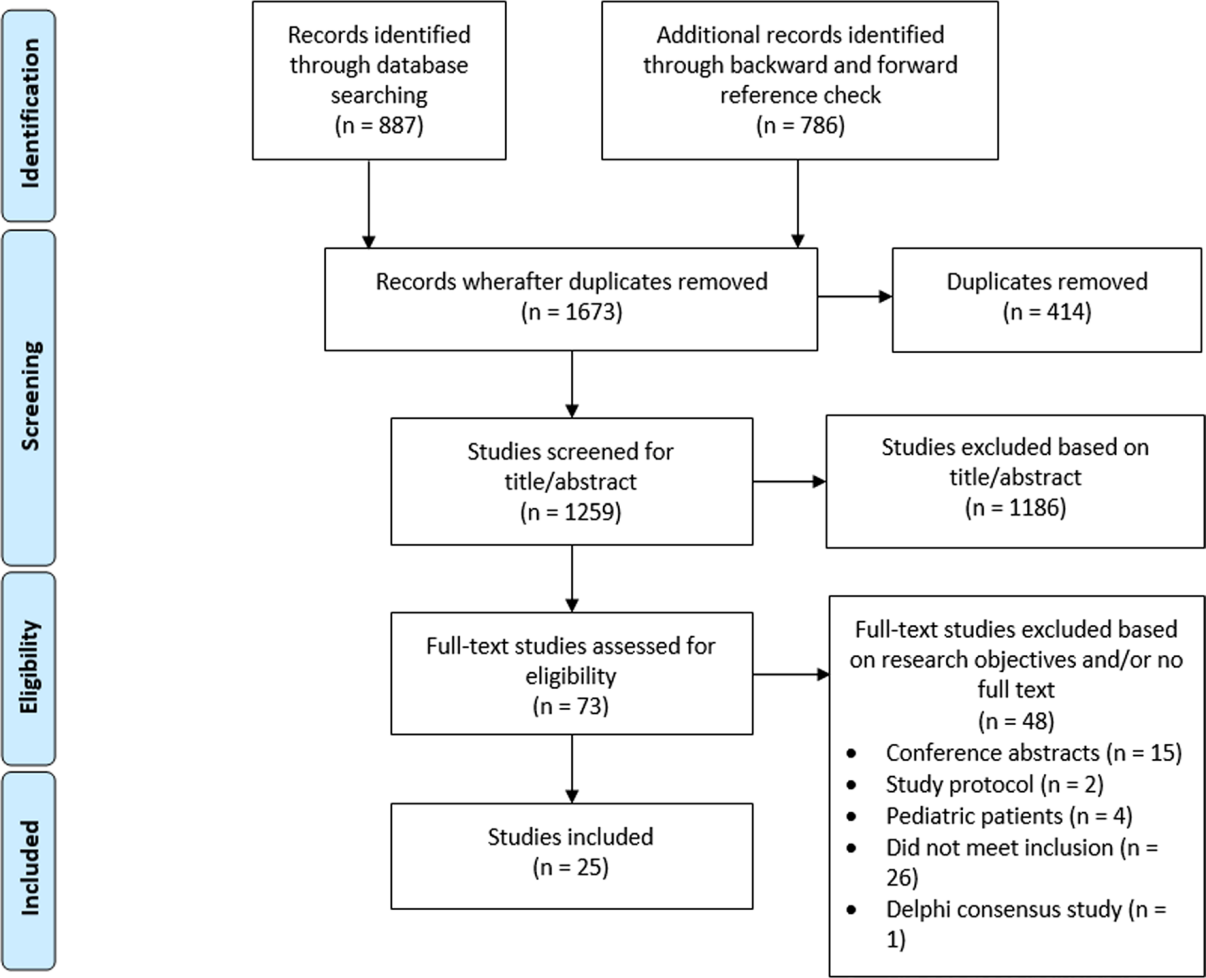
PRISMA flow chart of literature search and review process

Our search retrieved 1673 studies, from which 414 duplicates were excluded. Subsequently, from the remaining 1259 studies, 1186 were excluded; these did not describe PROMs data visualization (Fig. 1). Then, full-text articles from 73 eligible studies were assessed. From these, 47 were excluded based on inclusion and exclusion criteria, and/or because no full-text version was available (n = 15, all conference abstracts). Ultimately, 25 studies were included in this review for data extraction (Additional file 1: Table 2). After quality assessment following the CASP criteria, all studies had a positive score for half or more of the criteria (Additional file 1: Table 3).

**Fig. 2:**
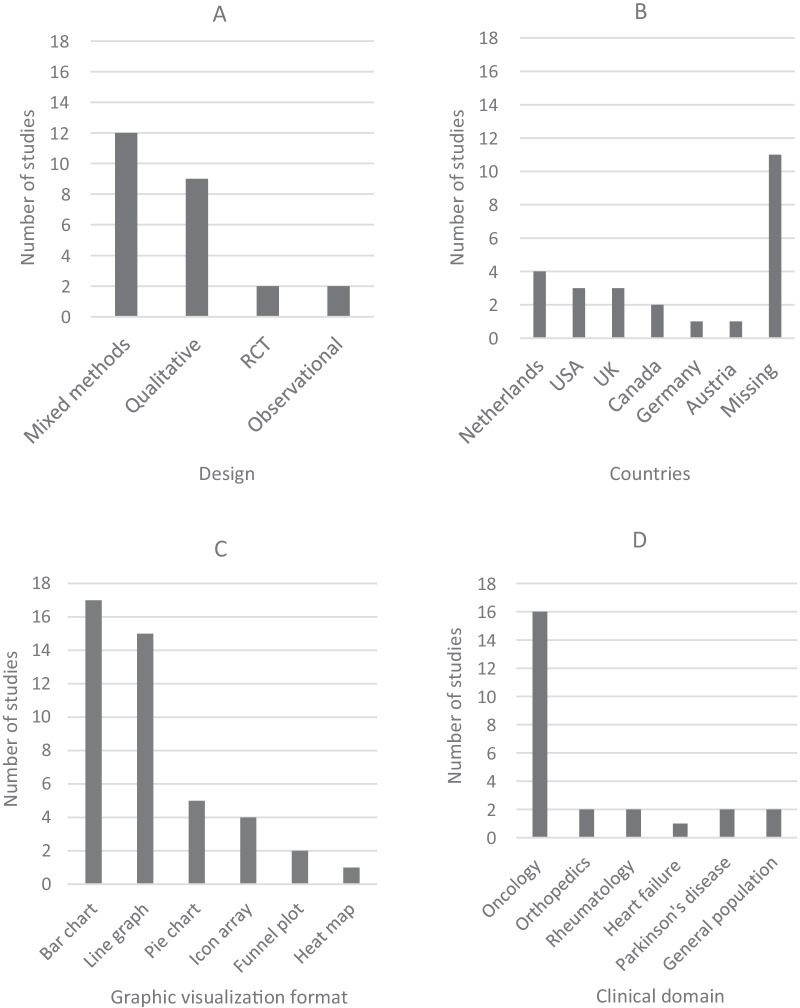
Studies included in this review were carried out using **a** different designs; **b** in different countries; **c** investigating different graphic visualization formats*, and **d** among different clinical domains. RCT: Randomized Controlled Trial; USA: United States of America; UK: United Kingdom. *Studies included multiple formats

**Fig. 3:**
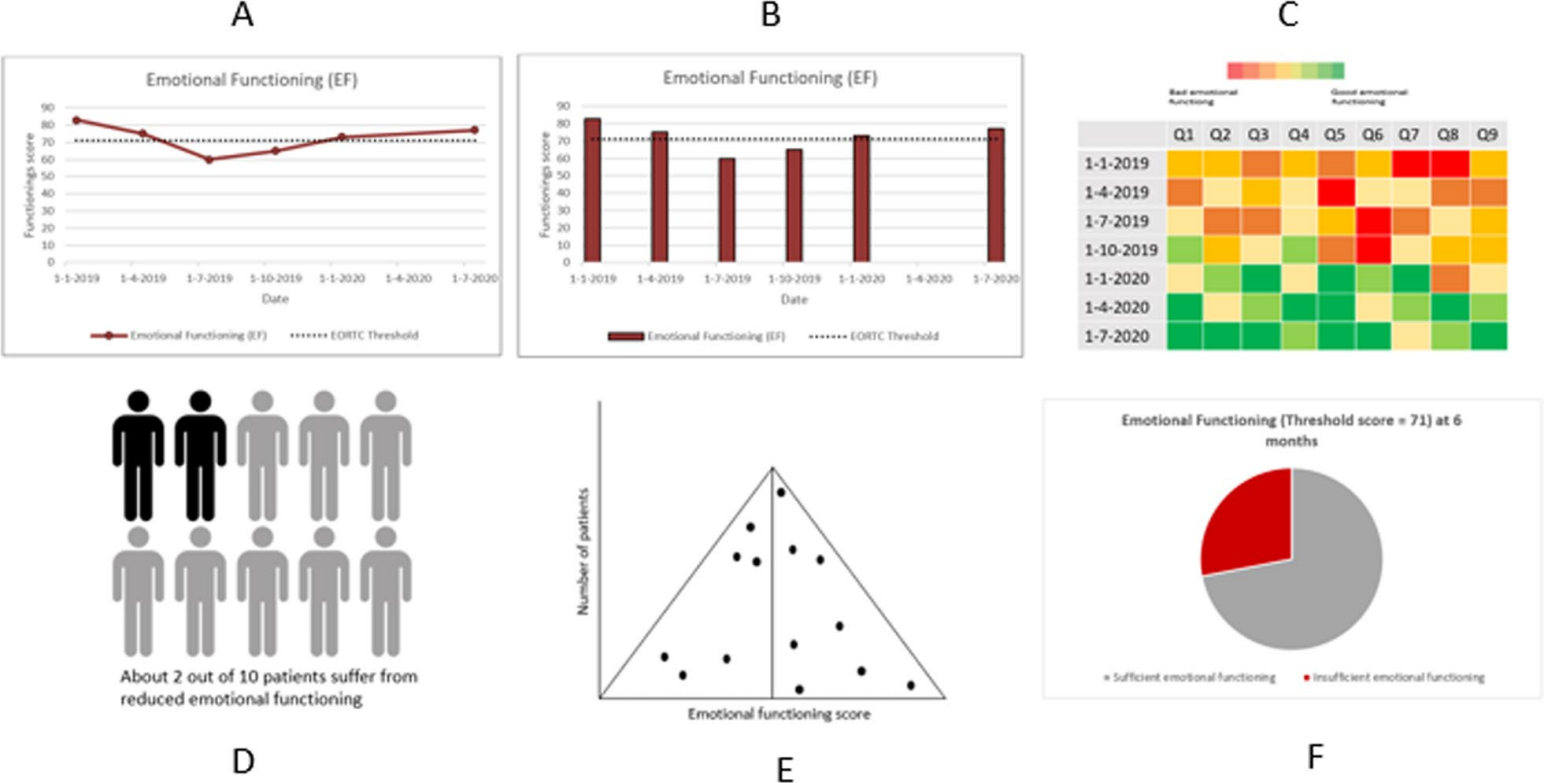
Different graphic visualization formats, presenting the domain of ‘emotional functioning’ as an example. Longitudinal: **a** line graph, including threshold line; **b** bar chart, including threshold line; **c** heat map. Cross-sectional: **d** icon array; **e** funnel plot; **f** pie chart

Most studies used either mixed methods design (n = 12), including human-centered design, or a qualitative design (n = 9), including interviews (Fig. 2). Sample sizes ranged from 8 (interview study) to 1,017 (survey study). Studies had been carried out in different clinical domains and in different countries, studying different graphic visualization formats and designs. The majority of studies included participants during or after treatment, whereas nine studies made use of hypothetical settings. PROMs data formats were either based on individual patient scores presented to patients (n = 17) and/or clinicians (n = 14), or based on mean group-reported data from for instance clinical trials that were presented to patients (n = 10) and clinicians (n = 8). The different graphic visualization formats that were studied are presented in Fig. 3. The results are presented according to the distinction between patients and clinicians, subdivided into preferences and interpretation accuracy.

### Individual level PROMs data—patients

An overview of the extracted data for patients on individual and group level is presented in Table 1.

**Table 1:** Summary of data extraction: visualization strategies and preferences, interpretation accuracy, comparators; use of PRO data on individual and group level, in patients

Author	Primary study goal	Study population	Presenting PROMs data		Visualization			
			Type of PROMs	What is presented?	Graphic visualisation format	Comparator	Outcomes of included studies preferences	Interpretation accuracy
**Individual level PROMs data visualization, patient**								
Brundage [16]	To investigate the interpretability of current PRO data presentation formats	N = 50 patients with variety of cancer diagnoses; N = 20 clinicians in active practice, Johns Hopkins Clinical Research Network (JHCRN)*	EORTC-QLQ-C30 scores	Individual scores and group means	Line graphs of mean scores	Previous scores	Simple line graphs for overall ease-of-understanding and usefulness	Patients accuracy ranged from 64–96% (line graphs questions)
					Tabulated scores		92% preferred formats displaying multiple time points	A graph trending down with better = higher scores was correctly interpreted by 96%. A graph trending down up with better = lower scores was correctly interpreted by 64%
					Bubble plots of scores at a point in time			
					Heat map			
Damman [14]	To investigate:	Interviews: patients with Parkinson's disease (N = 13) and clinicians (N = 14)	Not specified	Individual scores	Line graph	Patients with the same age, gender and disease duration	Bar chart is preferred (57.2%) compared to line graphs (42.3%)	What hindered easy comprehension: the use of a “higher = worse” directionality and comparative information of patients that are similar in terms of age, gender and disease progression
	(a) How patients and clinicians think about using PROMs during consultations;	Survey: patients (N = 115), the Netherland			Bar graph			Line and bar charts were interpreted most often correctly, compared with more “evaluative” formats like smileys and colors
	(b) For which purpose patients and clinicians use PROMs during consultations;				Line graph with comparative data over time (i.e. average scores of similar patients)			Individual PROMs scores over time were interpreted more often correctly when presented in a bar chart (87.8%) compared to a line graph (74.3%)
	(c) How patients interpret PROMs information presented in various formats							
Fischer [1]	To develop a PRO feedback report for mobile devices that is comprehensible and provides valuable information for patients after knee arthroplasty	Orthopedic patients (N = 8), Germany	Multiple (literature)	Individual scores	Text-based report and a graphical display (line graph, where scores are plotted over time, over a rainbow-colored background from red (bottom) to green (top) to visualize the grading of the individual scores)	Norm population	Short and condensed information using simple language (literature)	A text-based report is the least preferred but less susceptible to misinterpretation (literature)
			PROMIS (development)				An efficient way to present longitudinal PRO scores: graphs such as bar or line graphs (literature)	All participants correctly understood the line graph and were able to interpret the scores. Some needed some initial guidance on how to read a line graph
							Those (n = 3) in favor of graphs: easy and quick to get the relevant information from the line graph	The rainbow-colored background was understood by all participants
							The text-based (n = 2) version is easier to understand and most people are used to read short text messages	
Geerards [26]	To assess the impact of tailored multimodal feedback and computerized adapted testing (CAT) on user experience in HRQoL assessment using validated PROMs	N = 1386 participants from the general population, United Kingdom (UK)	World Health Organization Quality of Life assessment (WHOQOL)	Individual scores	Graphical only	N/A	Respondents thought the questionnaire with graphical and text-based feedback was more interesting compared with no feedback assessment, whereas providing only graphical feedback did not make the questionnaire more interesting	82.4% of patients thought the graphical feedback was accurate
					Graphical and adaptive text-based feedback			92.9% of patients thought the graphical feedback was clear
					Graphs: Separate horizontal bar charts for 4 domains			
					Text: What each domain reflects, how score corresponds to average scores, and what score might mean			
Grossman [27]	To identify the design requirements for an interface that assists patients with PRO survey completion and interpretation; to build and evaluate the interface of PROMs feedback	Interview: N = 13 patients with heart failure and N = 11 clinicians, study location or country was not described	Health IT Usability Evaluation Scale (Health-ITUES)	Individual scores	Small cards: Short sentence describing a severe symptom, which when clicked on provides textual educational information	N/A	Perceiving the mockup as useful and easy-to-use	Half of the participants failed to interpret the bar chart correctly, and even participants who could read it often required multiple attempts
		Usability testing: N = 12 patients with heart failure			Large cards:		Patients preferred visualizations with brief text descriptions	
					Symptom name and description, visual representation of its severity, and a link to textual educational information			
					Graph: Bar chart (lists patient’s symptoms from most to least severe, with symptom’s severity scores)			
Hartzler [6]	To conduct a HCD to engage patients, providers, and interaction design experts in the development of visual “PRO dashboards” that illustrate personalized trends in patients’ HRQoL following prostate cancer treatment	Focus groups (N = 60 patients)	Not specified	Individual scores	Pictographs	The dashboard compares patients’ trends with trends from “men like you” matched by default by age and treatment derived from a previously published prostate cancer cohort	Pictographs less helpful than bar charts, line graphs, or tables (P < .001)	Pictographs might reach patients with limited literacy
		N = 50 prostate cancer patients and N = 50 clinicians, study location or country was not described			Bar charts		Bar charts and line graphs are most preferred	Some patients expressed concern over inclusion of comparison scores without representation of data variability (e.g., confidence intervals, error bars), while others preferred simpler charts and graphs
					Line graphs			
Hildon [2]	To explore patients’ views of different formats and content of data displays of PROMs	N = 45 patients undergone or planning knee surgery in six focus groups, UK	Oxford Hip Score (OHS)	Individual scores	Different formats (table, bar chart, caterpillar and funnel plot)	N/A	Numerical tables lacked visual clarity	Representations of uncertainty were mostly new to the audience (numbers facilitated interpretation of uncertainty)
					Content (uncertainty displays, volume of outcomes, color, icons, and ordering)		Bar charts were liked because they were considered visually clear and facilitated appraisal at a glance, since it was a known format. But they do not give enough information	Traffic light colors were described as universally recognized
							Caterpillar plots were seen as visually clearer and to give more information but you would need to learn how to read them	Using colors consistently was important, as this enabled understanding across formats
							Funnel plots were difficult to read, had to learn how to read them	Stars were described as universally recognized and their interpretation did not require the ability to read
							Tables with icons were seen as accessible to the average person	The use of red and amber was thought to cause undue alarm while icons based on thumbs was seen as trivializing the issue
								Words (these were ‘at average’ ‘better’, ‘worse’, etc.) were liked because they were perceived as needing no personal interpretation
Izard [3]	To develop graphic dashboards of questionnaire responses from patients with prostate cancer to facilitate clinical integration of HRQoL measurement	N = 50 prostate cancer patients and N = 50 providers, USA	Expanded Prostate Cancer Index	Individual scores	Bar chart	Previous scores; ‘patients like me’	44% ranked bar chart dashboards as most preferred vs line graphs vs tables and pictographs	High reading scores for the table format
					Line graph			20% found pictograph too complicated (too many steps to interpret)
					Table that display HRQOL data in raw form			18% had difficulty disentangling facial expressions. Felt to be ‘‘too similar’’
					Facial expression pictograph			16% felt table to be easy to understand, 18% felt this format made HRQoL trends difficult to interpret
Kuijpers [4]	To investigate patients’ and clinicians’ understanding of and preferences for different graphical presentation styles for individual-level EORTC QLQC30 scores	N = 548 cancer patients in four European countries and N = 227 clinicians, the Netherlands	EORTC QLQ-C30	Individual scores	Bar chart with color	The preferred comparison group was one’s own previous results (40.9%)	39% preferred colored bar charts, over heat maps (20%) and colored line graphs (12%)	Objective understanding did not differ between graphical formats
					Bar chart without color			
					Line graph with color			
					Line graph without color			
					Heat map			
Liu [28]	To develop Rheumatoid Arthritis (RA) ‘dashboard’ that could facilitate conversations about PROs and is acceptable to a wide range of patients, including English and Spanish speakers, with adequate or limited health literacy	N = 25 RA patients and N = 11 clinicians from two academic rheumatology clinics, California	(1) Clinical Disease Activity Index (CDAI)	Individual scores	Line graph	Previous scores	Preference for more detailed information and more complex design in the adequate health literacy groups, but this preference was expressed by some limited health literacy participants as well	Several, particularly in the limited health literacy groups, did not notice or understand the longitudinal nature of data from left to right nor the temporal connection between the different graphic elements
			(2) Patient-Reported Outcomes Measurement Information System (PROMIS)-physical function scale					A few patients misinterpreted the line drawn between two data points to mean information from between the visits
			(3) Pain score					
Loth [28]	To investigate patients’ understanding of graphical presentations of longitudinal EORTC QLQ-C30 scores	N = 40 brain tumor patients, Austria	EORTC QLQ-C30	Individual scores	Colored bar chart	Previous scores	N/A	Objective correct answers about overall change was between 74.4% (fatigue) and 90.0% (emotional functioning)
x						Thresholds based on reference population		Difficulties with correct interpretation of different directionality of the symptom and functioning scales
						Values below/above a predefined threshold for clinical importance were given as green (clinically unimportant) or red (clinically important) bars. Thresholds for clinical importance were distribution-based		The meaning of color-coding to highlight clinically important problems was answered correctly by 100% of patients (physical function and pain), and 92.5% (emotional function and fatigue)
								90% of the patients reported that the graphs (overall change) were “very easy” or “rather easy” to understand (subjective understanding)
Oerlemans [5]	To investigate whether patients with lymphoma wished to receive PRO feedback, including the option to compare their scores with those of their peers, and how this feedback was evaluated	Lymphoma patients (N = 64), the Netherlands	EORTC-QLQ-C30 + item tingling in hands or feet	Individual scores	Bar chart	Previous scores	Respondents had a slight preference for bar charts	1 patient had trouble understanding the colors of the PRO feedback at first, but after looking for a second time it became clear
			Hospital Anxiety and Depression Scale (HADS)		Line graph	Reference population:	Preferred dotted line over a solid line to indicate “your score” in the bar chart	
			Adapted Self-Administered Comorbidity Questionnaire			General population		
						Scores other lymphoma patients		
						Patients: The vast majority (94%) compared their scores with those of the lymphoma reference cohort and 64% compared their scores with those of the normative population without cancer, whereas 6% viewed only their own scores		
Ragouzeos [25]	To develop a “dashboard” for RA patients to display relevant PRO measures for discussion during a routine RA clinical visit	Patients with rheumatology (N = 45) and providers (N = 12), USA	Not specified	Individual scores	Prototype PRO dashboard (on paper)	N/A	Important to show progress over time	Adding simple iconography and brief definitions of terms to the design helped patients understand which information the measured represented
							A longitudinal line graph with coloring helped patients see their measures as a process instead of a moment in time	
Smith [18]	To improve formats for presenting individual-level PRO data (for patient monitoring) and group-level PRO data (for reporting comparative clinical studies)	N = 40 clinicians in active practice and N = 39 patients diagnosed with cancer ≥ 6 months previously, not currently receiving chemotherapy/radiation or within 6 months of surgery, from JHCRN*	Not specified	Individual scores	Line graphs	Previous scores	N/A	Ease-of-understanding ratings were high for all formats, with median ranges from 9–10
					Pie charts			
					Bar charts			
					Icon array			
Snyder [34]	To test approaches for presenting PRO data to improve interpretability	N = 627 cancer patients/survivors, N = 236 oncology clinicians, and N = 250 PRO researchers for survey, from JHCRN*	Not specified	Individual scores	3 line-graphs:	Previous scores	N/A	82–99% correctly responded across directionality items
		N = 10 patients and N = 10 clinicians for interviews			(1) Green-shaded normal range			74–83% correctly identified domains that changed > 10 points
					(2) Red-circled possibly concerning scores			53–86% accurately identified possibly concerning scores
					(3) Red threshold-lines between normal and concerning scores			Red circles were interpreted more accurately than green shading
								Higher = better were interpreted more accurately versus higher = more
								Threshold-line significantly more likely to be rated “very” clear and most useful compared with green shading and red circles
**Group level/aggregated PROMs data visualization, patients**								
Brundage [12]	To explore patients' attitudes toward, and preferences for, 10 visual and written formats for communicating Health Related Quality of Life (HRQoL) information	N = 14 men and N = 19 women with variety of cancer diagnoses, post treatment ≥ 6 months earlier, Canada	PRO results from hypothetical clinical trial (cross-sectional, longitudinal)	Group mean scores	Mean HRQL scores:	Two treatments	Line graphs were preferred, because of their relative simplicity and straightforward layout	N/A
					Trends in text		Decrease in preferences for line graphs when error bars around the mean are presented	
					Mean scores			
					Mean scores with SD			
					Text			
					Change mean > 6 months			
Brundage [30]	To determine which formats for presenting HRQoL data are interpreted most accurately and are most preferred by patients	Patients with variety of cancer diagnosis, previously treated (N = 198), Canada	PRO results from hypothetical clinical trial (cross-sectional, longitudinal)	Group mean scores	Two treatments	N/A	Line graphs were preferred, due to high ease of interpretation and perceived helpfulness	Line graphs most often interpreted correctly (98%), most easy to understand, and most helpful (all p < 0.0001)
								Format type, participant age and education independent predictors of accuracy rates
Brundage [16]	To investigate the interpretability of current PRO data presentation formats	N = 50 patients with variety of cancer diagnoses; N = 20 clinicians in active practice, from JHCRN*	EORTC-QLQ-C30 scores	Individual scores and group means	Line graph means over time	Two treatments	Simple line graphs were preferred, since they have a high ease-of-understanding and usefulness	Accuracy ranged from 36% (cumulative distribution function question) to 100% (line graph with confidence intervals question)
					Line graph with norms		Line graphs are straightforward and clear	Patients tented to find normed scores, p-values and confidence intervals confusing
					Line graph with confidence intervals		87% of patients preferred formats displaying multiple time-points	
					Bar chart of average changes			
					Bar chart with			
					definition (improved, stable, worsened)			
					Cumulative distribution functions			
Damman [14]	To investigate:	Interviews: patients with Parkinson's disease (N = 13) and clinicians (N = 14)	Not specified	Individual scores	Line graph with results of 2 treatment options	Patients with the same age, gender and disease duration	56% of patients found line graphs most useful	Line graph showing results of two treatment options resulted in decisions reflecting adequate comprehension of information
	(a) How patients and clinicians think about using PROMs during consultations;	Survey: patients (N = 115), the Netherlands			Bar chart with results of 2 treatment options		47% of patients found bar charts most useful	
	(b) For which purpose patients and clinicians use PROMs during consultations;				Bar chart with performance of 2 providers		43% of patients found information with performance of two providers useful	
	(c) How patients interpret PROMs information presented in various formats							
McNair [32]	To assess patients’ understanding of multidimensional PROs in a graphical format	Patients with esophageal and gastric cancer (N = 132), UK	Semi-structured interviews	Mean scores	Line graphs:	Two treatments	N/A	87% of patients accurately interpreted multidimensional graphical PROs from two treatments
					(1) Treatment changes in a single PRO over time			81% of patients was able to interpret graph 4 correctly
					(2) Different PRO, reversed direction of treatment			67% of patients was able to integrate information from two graphs together
					(3) Divergent and convergent PROs			
					(4) Divergent and convergent PROs over 18 months			
Smith [18]	To improve formats for presenting individual-level PRO data (for patient monitoring) and group-level PRO data (for reporting comparative clinical studies)	N = 40 clinicians in active practice and N = 39 patients diagnosed with cancer ≥ 6 months previously, not currently receiving chemotherapy/radiation or within 6 months of surgery, from JHCRN*	Not specified	Individual scores, proportional data	Line graphs	Previous scores	55% of patients preferred pie charts	N/A
					Pie charts		25% of patients preferred icon arrays	
					Bar charts		20% of patients preferred bar charts	
					Icon array		45% of patients preferred formats with an asterisk indicating important differences	
Tolbert [29]	To identify the association of PRO score directionality and score norming on a) how accurately PRO scores are interpreted and b) how clearly they are rated by patients, clinicians, and PRO researchers	N = 629 patients (various oncologic diagnoses, N = 139 oncology clinicians, and N = 249 PRO researchers, conducted at the Johns Hopkins Clinical Research Network (JHCRN)*	Two treatments	Mean scores	Line graph 3 versions:	Two treatments	84% of patients rated “Better” formatted line graphs most often as “very clear” or “somewhat clear”	56% of patients answered questions correctly for “better” formatted lines, compared to 41% for “more” and 39% for “normed” graphs
					(1) Lines going up indicating “better” outcomes			The normed value confused patients
					(2) Lines going up indicating “more” (better for function domains, worse for symptoms). (3) Lines “normed” to a general population			
Tolbert [20]	To identify best practices for presenting PRO results expressed as proportions of patients with changes from baseline (improved/ stable/ worsened) for use in patient educational materials and decision aids	N = 629 patients (various oncologic diagnoses, treated), N = 139 oncology clinicians, and N = 249 PRO researchers, conducted at the Johns Hopkins Clinical Research Network (JHCRN)*	Two treatments	Proportions	Pie chart	Two treatments	Preferred pie charts: these were easy to read and enabled obtaining information quickly. Rated the clearest for communicating proportions changed from baseline	Patient’s accuracy was highest for pie charts and icon arrays
					Bar chart		Noted helpful aspects of bar charts: “Side by side comparisons are much easier to read and comprehend”	Bar graphs were less accurately interpreted than pie charts and icon arrays
					Icon array			Icon arrays would be easy to understand for patients
Van Overveld [19]	To investigate the preferences of receiving feedback between stakeholders	N = 37 patients, medical specialists, allied health professionals and health insurers in the Netherlands	Audit data on professional practice and health care outcomes	National average scores	Bar graph	National average scores	Patients preferred both a pie chart and a bar chart	Give feedback with average national scores
					Pie chart		Patients prefer a figure over plain text	National average scores on indicators of more interest for patient organizations and professionals
					Line graph			
					Point graph			
					Area graph			
					Box plot			
					Kaplan- Meier graph			
					Funnel plot			

Definitions: individual level PROMs data—The patient’s perspective on their health status; Group level PROMs data—Aggregated PROMs scores collected in clinical studies or trials

CDAI—Clinical Disease Activity Index; EHR—Electronic Health Record; EORTC-QLQ-C30—European Organization for Research and Treatment of Cancer Quality of life questionnaire Core 30; HADS—Hospital Anxiety and Depression Scale; HCD—Human Centered Design; HRQoL—Health-Related Quality of Life; HUI—Health Utility Index; Health-ITUES—Health IT Usability Evaluation Scale; JHCRN—Johns Hopkins Clinical Research Network; N/A—Not Applicable; PRO(s) —Patient Reported Outcome(s); PROMs—Patient Reported Outcome Measurements; PROMIS—Patient-Reported Outcomes Measurement Information System; QoL—Quality of Life; REALM-SF—Rapid Estimate of Adult Literacy in Medicine Short Form; SD—Standard Deviation; WHOQOL—World Health Organization Quality of Life

^*^JHCRN—Johns Hopkins Clinical Research Network: A consortium of academic and community health systems in the US mid-Atlantic with clinics outside the USA as well

#### Preferences

From the 25 studies included in this study, 12 assessed patients’ preferences. Patients appreciated that their individual PROMs scores were presented visually over time [16, 25]. Six studies reported that patients preferred bar charts over other graphic visualization formats for reporting longitudinal PROMs data [1, 3–6, 14]. Bar charts were ‘visually clear’, and facilitated ‘appraisal at a glance’, since bar charts are a generally well-known format for presenting data [2]. Five studies reported that line graphs were preferred as well [1, 6, 15, 16, 25]. Patients experienced line graphs as ‘easy to understand’ and ‘quick to retrieve relevant information from’ regarding their symptoms and well-being [1]. Furthermore, line graphs were preferred for overall ease-of-understanding and usefulness [16].

#### *Interpretation accuracy*

Eight studies assessed patients’ interpretation accuracy for visualizing individual PROMs data. Bar charts were more often interpreted correctly (87.8%) compared to line graphs (74.3%) [14]. According to Geerards et al. [26], a study conducted among 1386 participants of the general population, 92.9% of participants thought that bar charts were clear, and 82.4% thought bar charts were accurate. In a study by Loth et al. [17], among 40 patients, 90% reported that bar charts were “very easy” or “rather easy” to understand. Nevertheless, Grossman et al. [27] described that from 12 included patients, half of them failed to interpret bar charts correctly, and even patients who successfully read it, often required multiple attempts to do so. These participants were, however, introduced to bar charts for the first time [27]. One study reported that line graphs could be interpreted accurately by patients; nonetheless, an undefined proportion of patients needed initial guidance on how to read line graphs [1]. Liu et al. [28] reported that from 25 respondents (patients), several did not notice or understand the longitudinal nature of a line graph from left to right, particularly those with limited health literacy. Furthermore, a few patients misinterpreted a line drawn between two data points to mean information from between visits [28].

### Group-level or aggregated PROMs data—patients

The aim of presenting aggregated or mean PROMs scores to patients is to inform them about potential treatment outcomes, in order to support shared decision-making. In the included studies, aggregated PROMs data were mainly derived from clinical studies or RCTs. These were often presented to patients to (hypothetically) inform them about differences in outcomes between two treatments [12, 15, 20, 29–33].

#### Preferences

Seven studies assessed patients’ preferences for presenting aggregated outcomes over time. Four studies reported that patients preferred line graphs over bar and pie charts [12, 14, 16, 30]. Line graphs were preferred because of their relative simplicity, straightforward layout, and perceived helpfulness [12, 16, 30]. The preference for line graphs decreased when confidence intervals were presented [12]. To present proportional data, three studies reported that patients preferred pie charts [18–20]. Pie charts were easy to read and enabled obtaining information quickly [20]. Smith et al. [18] reported that of 39 patients, 55% preferred pie charts, compared to a 25% preference for icon arrays, and a 20% preference for bar charts. Van Overveld et al. [19] reported that among 37 patients, pie and bar charts were both preferred.

#### Interpretation accuracy

Seven studies assessed patients’ interpretation accuracy for presenting aggregated outcomes over time. In four studies, patients’ most often correctly interpreted line graphs [14, 16, 29, 30]. Interpretation accuracy ranged from 98–100% [16, 30]. In particular, 56% of patients correctly answered questions for “better” (i.e. higher score means better outcomes) formatted lines, compared to 41% for “more” (i.e. higher score means more symptoms), and 39% for “normed” (i.e. score as compared to reference scores) line graphs [29]. Line graphs showing results of two treatment options resulted in decisions that reflected adequate comprehension of the information in the graph [14]. One study reported that patients’ accuracy was highest for pie charts and icon arrays, compared to bar charts [20]. Icon arrays were easy to understand for patients as well [20].

### Individual level PROMs data—clinicians

An overview of the extracted data for clinicians on individual and group level is presented in Table 2.

**Table 2:** Summary of data extraction: visualization strategies and preferences, interpretation accuracy, comparators; use of PRO data on individual and group level, in clinicians

Author	Primary study goal	Study population	Presenting PROMs data		Visualization		Outcomes of included studies	
			Type of PROMs	What is being presented?	Graphic visualisation format	Comparator	Preferences	Interpretation accuracy
**Individual level PROMs data visualization, clinicians**								
Brundage [16]	To investigate the interpretability of current PRO data presentation formats	N = 50 patients with variety of cancer diagnoses; N = 20 clinicians in active practice, from Johns Hopkins Clinical Research Network (JHCRN)*	EORTC-QLQ-C30 scores	Individual scores and group means	Two line graphs:	Previous scores	Preference for line graphs: overall ease-of-understanding and usefulness	Interpretation accuracy was high across formats
					Higher = better functioning		90% of clinicians preferred formats displaying multiple time-points vs single time-points	Inconsistency between whether higher scores were better or worse contributes to incorrect accuracy (Uptrend lines intuitively signify improvement of understanding)
					Higher = more symptoms		Tabulated scores were considered boring but straightforward	
					Bubble plot			
					Heat maps			
Brundage [37]	To evaluate the interpretation accuracy and perceived clarity of various strategies for displaying clinical trial PRO findings	Oncology clinicians (N = 233) and PRO researchers (N = 248), from JHCRN*	PRO results from hypothetical clinical trial (cross-sectional, longitudinal)	Longitudinal individual data, proportions	Bar chart	Line graph also normed against general population	Not one approach for either longitudinal data or proportions changed is universally appealing, nor is free of misinterpretation errors	Line graph:
					Pie chart			More likely to be interpreted correctly “better” vs “normed” graphs (p = 0.04)
					3 Line graphs:			No differences between “better” and “more”
					1) Higher = “better” functioning;			Regardless of graph type and version, adding asterisks for clinical significance and confidence limits did not contribute to better interpretation accuracy
					2) Higher = “more” symptoms;			Bar chart/pie chart:
					3) “Normed” against general population			Respondents less likely to make interpretation errors with pie vs bar charts (p < 0.001)
								Odds of selecting an “incorrect” treatment significantly lower in pie charts compared to bar charts
								Clarity ratings did not differ between formats
Damman [14]	To investigate:	Interviews: patients with Parkinson's disease (N = 13) and clinicians (N = 14)	Not specified	Individual scores	Line graph	Patients with the same age, gender and disease duration	Strong preference for individual PROMs data over time	Individual PROMs scores with comparative data of similar patients were found useful, some expressed doubts
	(a) How patients and clinicians think about using PROMs during consultations;	Survey: patients (N = 115), the Netherlands			Bar graph		Line and bar graphs	
	(b) For which purpose patients and clinicians use PROMs during consultations;				Line graph with comparative data over time (i.e. average scores of similar patients)		Scores from repeated measurements over time	
	(c) How patients interpret PROMs information presented in various formats						Multiple individual quality of life, rather than one overall quality of life score	
							Identified the possibility to use aggregated PROMs scores as evidence for treatment options	
Grossman [27]	To identify the design requirements for an interface that assists patients with PRO survey completion and interpretation; to build and evaluate the interface of PROMs feedback	Interview: N = 13 patients with heart failure and N = 11 clinicians, study location or country was not described	Health IT Usability Evaluation Scale (Health-ITUES)	Individual scores	Small cards: Contains a short sentence to describe a severe symptom, which when clicked on provides textual educational information	N/A	Perceiving the mockup as useful and easy-to-use	Two providers reported that PROs might reduce their cognitive load
		Usability testing: N = 12 patients with heart failure			Graph:		Preference for tracking symptoms over time	
					Bar chart that lists the patient’s symptoms from most to least severe and displays each symptom’s severity score			
					Large cards:			
					Displays a symptom name, its two-sentence description, a visual representation of its severity, and a link to textual educational information			
Hartzler [36]	To share lessons learned from engaging clinicians to inform design of visual dashboards	Clinicians: N = 12 for interviews, N = 40 for surveys and consensus meeting, N = 9 for user testing, study location or country was not described	Not specified	Individual scores	PRO data needs appear to differ for health care providers and administrative staff as key target users	N/A	Participants liked viewing trends over time	Value was found in developing meaningful ways to report on this new source of data
					The functional prototype has 3 components:		Participants found the views to provide a useful basis for comparison	In addition to the information buttons provided on “how to interpret this chart,” clear labels are needed, such as on chart axes
					(1) An “At a glance” screen providing a simple data overview of PROs data			
					(2) An “Analyze” screen providing a data view the user can filter			
					(3) A “Data quality” screen			
Hartzler [6]	To conduct a HCD to engage patients, providers, and interaction design experts in the development of visual “PRO dashboards” that illustrate personalized trends in patients’ HRQoL following prostate cancer treatment	Focus groups (N = 60 patients)	Not specified	Individual scores	Pictographs	The dashboard compares patients’ trends with trends from “men like you” matched by default by age and treatment derived from a previously published prostate cancer cohort	Pictographs less helpful than bar charts, line graphs, or tables (P < .001)	Pictographs might reach patients with limited literacy
		N = 50 prostate cancer patients and N = 50 clinicians, study location or country was not described			Bar charts		Preferred bar charts and line graphs	Some participants, both patients and providers, expressed concern over inclusion of comparison scores without representation of data variability (e.g., confidence intervals, error bars), while others preferred simpler charts and graphs
					Line graphs			
Izard [3]	To develop graphic dashboards of questionnaire responses from patients with prostate cancer to facilitate clinical integration of HRQoL measurement	N = 50 prostate cancer patients and N = 50 providers from Seattle, USA	Expanded Prostate Cancer Index	Individual scores	Bar chart	Previous scores; ‘patients like me’	No universally preferred dashboard format: 30% preferred tables, 34% preferred bar charts, and 34% preferred line graphs	Helpfulness and confidence ratings varied among dashboard format. Pictographs had the lowest helpfulness compared with table, bar, and line graph formats
					Line graph			
					Table that display HRQOL data in raw form			
					Facial expression pictograph			
Jagsi [35]	To investigate practicing oncologists view on incorporating routine collection of PROs into cancer care	N = 17 oncologists, USA	Edmonton Symptom Assessment System	Individual scores	Bar chart	Previous scores	Ability to track symptoms over time and effect of intervention	Keep it simple: limit number of symptoms
								Link number scale to narrative
Kuijpers [4]	To investigate patients’ and clinicians’ understanding of and preferences for different graphical presentation styles for individual-level EORTC QLQC30 scores	N = 548 cancer patients in four European countries and N = 227 clinicians, the Netherlands	EORTC QLQ-C30	Individual scores	Bar chart with color	The preferred comparison group was one’s own previous results (40.9%)	Medical specialist:	Medical specialist:
					Bar chart without color		Heat map (46%)	Objective understanding of 78%
					Line graph with color		Nurses:	Nurses:
					Line graph without color		Bar chart (non-colored) and heat map (32%)	Objective understanding of 74%
					Heat map			85% of all HCP’s indicated that the graphs were (easy) to understand, not differing between professions or graphical presentation styles
								Uniformity in scoring direction would be preferred
								A significant difference for overall change scores, with the non-colored bar charts being interpreted correctly more often than the other graphical displays
Ragouzeos [25]	To develop a “dashboard” for RA patients to display relevant PRO measures for discussion during a routine RA clinical visit	Patients with rheumatology (N = 45) and providers (N = 12), USA	Not specified	Individual scores	Prototype PRO dashboard (on paper)	Previous scores	N/A	Information needs to be clearly focused on what is most essential
								Important to show progress over time
Santana [33]	To describe the process, feasibility and acceptability of use of the Health Utilities Index (HUI) in routine clinical care	Pre- and post-heart and -lung transplant patients (N = 151), Canada	Health Utilities Index (HUI)	Individual scores	HUI score card, using a color-coded system	Pre- and post-treatment scores	N/A	Clinicians did not need much time to understand the use of the HUI score card
								Clinicians developed their own way of using the information over time
Smith [18]	To improve formats for presenting individual-level PRO data (for patient monitoring) and group-level PRO data (for reporting comparative clinical studies)	N = 40 clinicians in active practice and N = 39 patients diagnosed with cancer ≥ 6 months previously, not currently receiving chemotherapy/radiation or within 6 months of surgery, from JHCRN*	Not specified	Individual scores, proportional data	Line graphs	Previous scores	75% preferred the line graph	Ease-of-understanding ratings were high for all formats
					Pie charts			Directional inconsistency emerged as an interpretation challenge
					Bar charts			Format interpretation challenges included explaining the meaning of scores (i.e., whether scores are good/bad, what normal is)
					Icon array			
Snyder [34]	To test approaches for presenting PRO data to improve interpretability	N = 627 cancer patients/survivors, N = 236 oncology clinicians, and N = 250 PRO researchers for survey, from JHCRN*	Not specified	Individual scores	3 line-graphs:	Previous scores	N/A	The proportion responding correctly across the 4 directionality items ranged from 80 to 100%
		N = 10 patients and N = 10 clinicians for interviews			1) Green-shaded normal range			Red circles were interpreted more accurately than green shading
					2) Red-circled possibly concerning scores			Higher = better were interpreted more accurately versus higher = more
					3) Red threshold-lines between normal and concerning scores			Threshold-line significantly more likely to be rated “very” clear and most useful compared with green shading and red circles
**Group level/aggregated PROMs data visualization, clinicians**								
Brundage [16]	To investigate the interpretability of current PRO data presentation formats	N = 50 patients with variety of cancer diagnoses; N = 20 clinicians in active practice, from JHCRN*	EORTC-QLQ-C30 scores	Individual scores and group means	Line graph means over time	Two treatments (study arms)	90% of clinicians preferred formats displaying multiple time-points	Line graphs contributed to overall ease-of-understanding and usefulness
					Line graph with norms		Preference for line graphs of normed sores or with confidence intervals	Normed scores provided basis for comparison beyond two treatments, p-values and confidence intervals were particularly important for publications
					Line graph with confidence intervals		Some preference for bar charts to compare treatments	Cumulative distributing function confusing and difficult to interpret
					Bar chart of average changes			Inconsistency between whether higher scores were better or worse contributes to incorrect accuracy
					Bar chart with definition (improved, stable, worsened)			
					•Cumulative distribution functions			
Damman [14]	To investigate:	Interviews: patients with Parkinson's disease (N = 13) and clinicians (N = 14)	Not specified	Individual scores	Line graph with results of 2 treatment options	Patients with the same age, gender and disease duration	Identified the possibility to use aggregated PROMs scores as evidence for treatment options	N/A
	(a) How patients and clinicians think about using PROMs during consultations;	Survey: patients (N = 115), the Netherlands			Bar chart with results of 2 treatment options		Aggregated PROMs scores for provider options could be useful, but would not be used much in clinical practice	
	(b) For which purpose patients and clinicians use PROMs during consultations;				Bar chart with performance of 2 providers			
	(c) How patients interpret PROMs information presented in various formats							
Liu [28]	To develop Rheumatoid Arthritis (RA) ‘dashboard’ that could facilitate conversations about PROs and is acceptable to a wide range of patients, including English and Spanish speakers, with adequate or limited health literacy	N = 25 RA patients and N = 11 clinicians from two academic rheumatology clinics, California (USA)	(1) Clinical Disease Activity Index (CDAI)	Individual scores	Line graph	Aggregated clinical data	A dashboard is a potential method for aggregating data from various sources	A ‘snapshot’ of relevant information for a particular patient would make HCP’s own medical decisions easier
			(2) Patient-Reported Outcomes Measurement Information System (PROMIS)-physical function scale				Clinicians were very interested in customizing the dashboard to their own needs and recommended that it can be designed to present information that is more detailed	
			(3) Pain score					
Smith [18]	To improve formats for presenting individual-level PRO data (for patient monitoring) and group-level PRO data (for reporting comparative clinical studies)	N = 40 clinicians in active practice and N = 39 patients diagnosed with cancer ≥ 6 months previously, not currently receiving chemotherapy/radiation or within 6 months of surgery, from JHCRN*	Not specified	Individual scores, proportional data	Line graphs	Average changes	For proportional data formats: pie charts (70%)	Median ease-of-understanding ranged from 6.5 to 8
					Pie charts		Few clinicians (10%) preferred bar charts	Mixed feelings about indications of clinical significance in terms of having p-values in addition to confidence intervals and asterisks indicating important differences
					Bar charts		75% preferred the line graph	Directional inconsistency emerged as an interpretation challenge
					Icon array			Format interpretation challenges included explaining the meaning of scores (i.e., whether scores are good/bad, what normal is)
Tolbert [29]	To identify the association of PRO score directionality and score norming on a) how accurately PRO scores are interpreted and b) how clearly they are rated by patients, clinicians, and PRO researchers	N = 629 patients (various oncologic diagnoses), N = 139 oncology clinicians, and N = 249 PRO researchers, conducted at the Johns Hopkins Clinical Research Network (JHCRN)*	Two treatments	Mean scores	Line graph 3 versions:	Two treatments	“Better” formatted line graphs were rated most often as “very clear” or “somewhat clear” by all three groups (84% by patients, 81% by clinicians, and 85% by researchers)	Answers correct for “better”: 70%; “more”: 65%; “normed”: 65%
					(1) Lines going up indicating “better” outcomes		However, the range in the proportion rating each format “very clear” or “somewhat clear” was narrow: 77% to 85%	“More” line graph comments noted that up could mean different things, which could lead to errors
					(2) Lines going up indicating “more” (better for function domains, worse for symptoms). 3) Lines “normed” to a general population average of 50			“Better” line graph comments pointed out how changing the scale could result in interpretation errors as one must orient to the direction of the scale each time
Tolbert [20]	To identify best practices for presenting PRO results expressed as proportions of patients with changes from baseline (improved/ stable/ worsened) for use in patient educational materials and decision aids	N = 629 patients (various oncologic diagnoses, treated), N = 139 oncology clinicians, and N = 249 PRO researchers, conducted at the Johns Hopkins Clinical Research Network (JHCRN)*	Two treatments	Proportions changed	Pie chart	Two treatments	Preferred pie charts: these were easy to read and enabled obtaining information quickly	Clinician and researchers scored pie charts as the most accurately interpreted
					Bar chart		43% had positive feedback on icon arrays	In general, bar graphs were less accurately interpreted than pie charts and icon arrays
					Icon array		38% had positive feedback on bar charts	Noted helpful aspects of bar charts: “Side
								by side comparisons are much easier to read and comprehend”
van Overveld [19]	To investigate the preferences of receiving feedback between stakeholders	N = 37 patients, medical specialists, allied health professionals and health insurers in the Netherlands	Audit data on professional practice and health care outcomes	National average scores	Bar graph	National average scores	Preference for bar charts since they are easier to read	Box plots, Kaplan–Meier graphs and funnel plots give a less clear overview and are more difficult to interpret
					Pie chart		For survival and process indicators: Kaplan–Meier graphs and box plots	Find a balance between giving feedback and giving too much information
					Line graph			Give an overview of the results first, followed by the details
					Point graph			Present it that one can easily understand without explanation
					Area graph			
					Box plot			
					Kaplan- Meier graph			
					Funnel plot			

Definitions: individual level PROMs data—The patient’s perspective on their health status; Group level PROMs data—Aggregated PROM scores collected in clinical studies or trials

Abbreviations: CDAI—Clinical Disease Activity Index; EHR—Electronic Health Record; EORTC-QLQ-C30—European Organization for Research and Treatment of Cancer Quality of life questionnaire Core 30; HADS—Hospital Anxiety and Depression Scale; HCD—Human Centered Design; HRQoL—Health-Related Quality of Life; HUI—Health Utility Index; Health-ITUES—Health IT Usability Evaluation Scale; JHCRN—Johns Hopkins Clinical Research Network; N/A—Not Applicable; PRO(s) —Patient Reported Outcome(s); PROMs—Patient Reported Outcome Measurements; PROMIS—Patient-Reported Outcomes Measurement Information System; QoL—Quality of Life; REALM-SF—Rapid Estimate of Adult Literacy in Medicine Short Form; SD—Standard Deviation; WHOQOL—World Health Organization Quality of Life

^*^JHCRN—Johns Hopkins Clinical Research Network: A consortium of academic and community health systems in the US mid-Atlantic with clinics outside the USA as well

#### Preferences

Thirteen studies assessed clinicians’ preferences regarding visualization of PROMs scores from individual patients. In general, clinicians appreciated viewing PROMs scores repeated over time, in order to track their patients’ symptom experiences [14–16, 25, 35, 36]. Moreover, six studies showed that bar charts were most preferred when plotting longitudinal individual PROMs data [1, 2, 4, 5, 14, 17]. Furthermore, line graphs were preferred in four studies [1, 14–16, 29]. However, Brundage et al. [37] and Izard et al. [3] both stated that clinicians did not universally find one approach for longitudinal data as appealing or preferred. Contrastingly, a study among 227 health professionals by Kuijpers et al. [4] showed that the majority of medical specialists (46%) and nurses (32%) preferred heat maps to line graphs and bar charts.

#### Interpretation accuracy

Thirteen studies assessed clinicians’ interpretation accuracy of graphic visualization formats for individual PROMs level data. Multiple studies showed that clinicians’ interpretation accuracy was similar over different graphic visualization formats: both line graphs and bar charts were found easy to understand, and were interpreted accurately [3, 4, 18]. Hartzler et al. [36] reported that among twelve clinicians, respondents generally felt that both line graphs and bar charts provide a useful basis for comparison. Pictographs were reported to be the least helpful for clinicians, although clinicians stated these could be helpful for patients with limited literacy [3, 6]. Uniformity in directionality of scores could increase clinicians’ interpretation accuracy in different graph formats. However, Brundage et al. [37] found that adding asterisks for clinical significance and confidence limits around scores did not contribute to a better interpretation accuracy in clinicians.

### Group-level or aggregated PROMs data—clinicians.

#### Preferences

When presenting aggregated PROMs data, seven studies stated that clinicians mostly compared data between two treatments or compared scores to mean reference population scores. Brundage et al. [16] stated that for comparing treatments, 18 out of 20 clinicians preferred formats displaying multiple time-points, with the highest preference for line graphs with normed scores or confidence intervals, or bar charts. Smith et al. and Tolbert et al. [18, 29] describe a preference for line graphs, with over 75% of included clinicians preferring this graphical format; bar charts were less supported, as only 10% of 40 clinician participants preferred bar charts [18]. Van Overveld et al. [19] reported however that bar charts were preferred, since they were easy to read. To present proportional data, pie charts were most preferred in 3/8 studies [18, 20, 34]. To meet a variety of preferences, Liu et al. [28] found that a dynamic dashboard gave clinicians the opportunity to customize the formats to their own needs.

#### Interpretation accuracy

Six studies assessed clinicians’ interpretation accuracy on aggregated data. Line graphs usually contributed to the ease of understanding PROMs scores for clinicians [16, 29]. Additionally, pie charts with proportional data were most often interpreted accurately by clinicians, for example when pie charts presented a proportional change in outcomes compared to baseline [20]. Clinicians both endorsed and objected that p-values, confidence intervals, and normed scores could contribute to their interpretation accuracy of aggregated PROMs data [16, 18]. Furthermore, directional consistency, balancing information, and giving feedback, and clear labeling could improve interpretation accuracy [18, 19].

Throughout many of the included studies, challenges were described that may affect correctly interpreting visualized PROMs data, such as (1) Directional inconsistency, i.e. a *higher* functioning score means better health, but a *lower* symptom score means better health as well [5, 16, 17, 34, 37]; (2) Lack of standardisation rules for interpretation and visualization, that may cause interpretation inaccuracy [2, 25, 35]; (3) The need to designing multiple formats per target group, as no ‘one-size-fits-all’ solution in graphic visualisation exists for both patients and clinicians [3, 6, 28]; (4) The timing of providing feedback on PROMs visualization, as this affects assessment experience [14, 26], and (5) Patients ‘ opposition to PROMs use in clinical practice [19, 36]. We summarize the challenges and the proposed solutions for these challenges in Table 3.

**Table 3:** Challenges and factors for improvement to consider when implementing visual individual PROMs feedback in clinical practice

Challenges that may hinder graphic visualization format interpretation	Possible factors to improve graphic visualization format interpretation
**Patients and clinicians**	
Directional inconsistency in longitudinal data (i.e., sometimes higher scores can mean better or worse)	Make use of standard descriptive labels (consider using ‘better*’ instead of ‘normed**’ or ‘more***’ for describing directionality of scores) [34, 37]
	Preferred by 79% of patients and 90% of clinicians when concerning individual level PROMs data and 100% of clinicians when concerning group level PROMs data
	Consistent use of clear ratings: higher scores are always better results (i.e. in some frequently used PROMs, higher score are better when scores describe functioning, but lower score are better when symptom burden is described. This causes interpretation challenges) [37]
	Indicate with an arrow on the y-axis which direction means the score is better [16]
	Describe directionality by plain text that is understandable despite literacy or education level [5]
	Provide detailed information on the meaning of high and low score [17]
Interpretation accuracy of what exact PROMs information is represented in the graphic visualization format	Provide an instructive aid for patients and clinicians [2]
	Use simple iconography for demonstrating single PROMs values [25]
	Use brief definitions of different PROMs domains/values [25]
	Limit the number of presented symptoms per graphic visualization format [35]
No ‘one-size-fits-all’ solution	Make use of a dynamic dashboard, which can display multiple types of visualization strategies. Thereby, you provide users the ability to select a preferred format instead, including the ability to add or remove dashboard elements such as error bars and shading [3, 6, 28]
	Developing a clinic-based video tutorial for the dashboard to explain what is shown on the dashboard and how the patient and clinician can customize the dashboard to their needs [28]
**Patients**	
Interpretation accuracy of what exact PROMs information is represented in the graphic visualization format	Ask patients to prioritize their symptoms, to avoid an overload of information [35]
Timing of providing feedback on PROMs visualization	Provide feedback immediately after assessment, and before consultation, to significantly improve assessment experience when providing combined graphical and tailored text-based feedback [14, 26]
Patients ‘ opposition to PROMs use in clinical practice	Ask permission to the patient to receive their own results and/or the results of the general population [19]
	Provide information so patients know what PROMs data might show and how their practice might change [36]
	Tell patients that data is trustworthy and are handled confidentially [19, 36]
	Do not provide anonymous feedback [19]
	Visualize as transparently as possible what type of care is delivered [19]
**Clinicians**	
Interpretation accuracy of what exact PROMs information is represented in the graphic visualization format	Eliminate comparison groups or inform comparison group scores with confidence intervals or error bars [3], to better counsel the patients about their score (makes it easier to understand)
	Link the PROMs outcome scores (scale in the graphic visualization format) to the meaning of the narrative (i.e.; tell the patient that a higher score on the scale means better functioning) [35]

PROMs: patient reported outcome measures

^*^’Better’ is defined as higher scores indicating “better” outcome

^**^’Normed’ is defined as normed to the general U.S. population

^***^’More’ is defined as higher scores indicating “more” of what was being measured

## How to distinguish clinically relevant PROMs scores

Clinically alarming scores were put in perspective by comparing current scores to the patients’ previous scores or to norm population scores. Ten studies described strategies to distinguish clinically relevant scores in practice [3–5, 16–18, 35]. These studies were conducted internationally among patients with different cancer diagnoses. Most frequently used comparator scores were the patients’ own previous PROMs data [3–5, 16–18, 35]. Additionally, four studies used a norm population (i.e.; patients with the same disease) to determine the clinical relevance of scores [1, 5, 19, 37]. Patients pointed out that the comparison of their own data with the scores from ‘patients like them’ (i.e. same sex, and age) was most valuable: it puts the patients functioning in perspective of what is regarded ‘normal’ [5].

Studies showed different methods of applying color to highlight scores, including: visualizing clinically non-alarming scores in green and clinically alarming scores in red [17]; red and green shading to show undesirable and desirable score ranges, respectively [4, 5, 18, 25]; a background fading from red (bottom) to green (top) [1]; traffic light colors (i.e. green, orange, red) [2].

Smith et al. [18] reported that 74% of patients and 80% of clinicians preferred green shading of non-alarming scores or red shading of alarming scores. Hildon et al. [2] reported that traffic light colors could improve interpretation accuracy across different graphic visualization formats, since these are universally recognized. Loth et al. [17] reported that 93% of the studied patients correctly interpreted the meaning of traffic light colors. Nonetheless, PROMs visualization strategies must include a detailed explanatory legend of the meaning and interpretation of colors and scores [4].

Other visualization strategies included the use of red circles around important scores—these were interpreted more accurately than green shading [34]. Furthermore, threshold lines across score bars were used to indicate whether scores are better or worse than threshold scores. These visualization techniques pleased 69% of patients and 70% of clinicians [17, 18]. Contrastingly, Snyder et al. [34] reported that a threshold-line was significantly more often rated as ‘very clear’, and most useful compared to green shading, and red circles. In more detail, another study found that a dotted threshold line was preferred over a solid threshold line to indicate alarming scores in bar charts [5]. Lastly, exclamation points can be used to indicate possibly concerning score changes, which was the preferred method for 79% of 39 patients and 40% of 40 clinicians [18].

## Discussion

This systematic review included 25 studies in different healthcare settings throughout Europe, the US, and Canada, that reported about preferences and interpretation accuracy of patients and clinicians for the visualization of PROMs scores. Very few graphical visualization formats for presenting PROMs data could be identified. Overall, a limited amount of literature was found on this matter, which was summarized as follows. For individual level PROMs data, patients and clinicians preferred line graphs and bar charts, since they were considered visually clear. Bar charts were most often interpreted correctly by patients, while clinicians had high interpretation accuracy across all graph formats. For presenting group level PROMs data, pie charts and line graphs were preferred: patients most often interpreted line graphs correctly; for proportional data, clinicians most often accurately interpreted pie charts. To guide clinical interpretation by distinguishing clinically relevant scores, PROMs scores were most often compared to patients’ previous scores, followed by comparison to mean norm population scores. Here, correct interpretation can be supported by highlighting patients’ clinically alarming scores with colors, and by using threshold lines across score bars or lines, or circles around alarming scores.

Furthermore, we looked into the challenges that may hinder graphic visualization format interpretation. An underlying cause of incorrect interpretation of graphs may be the lack of standardization in rules for interpretation; variability exists in score directionality (e.g. higher scores can either indicate better or worse outcomes), and scaling (e.g. scores ranging from 0 to 100 indicating the worst-to-best possible scores, or scores ‘normed’ to a defined population). Furthermore, meaningful interpretation of PROMs scores is complicated by the way the statistical and clinical significance of the findings (i.e. thresholds to distinguish clinical importance) are addressed [16, 18, 38, 39]. Therefore, effort must be made to present PROMs scores to patients and clinicians more accurately by: improving directional consistency by making use of standard descriptive labels [34, 37], clear label ratings (i.e. consistent scales ranging from 0–100 [37]), detailed information on the meaning of high and low scores [17], simple iconography [25], and brief definitions to understand what the PROMs scores represent [25]. Furthermore, it was suggested is to visualize only a limited number of symptoms, and to ask patients to prioritize the symptoms they want feedback on [35]. Afore mentioned factors to guide clinical interpretation of graphic visualization formats of PROMs data, were identified as well in a Delphi-consensus study by Snyder et al. [15]. In this study, a panel including 15 doctor or nurse clinicians, 10 participants who identified as patient or caregiver advocates, 12 researchers, and 6 members of journal editorial boards, were asked to review data display issues, and give their perspectives on these issues to develop consensus statements. The authors conclude that implementation of graphic visualization formats of PROMs data have enormous potential to promote patient-centred care, however, it is critical that patients and clinicians understand what PROMs scores mean. More specifically, they recommended to use exceptionally clear labelling, titling, and other annotations to address potential confusion in direction of scores, and warn for mixing score direction in a single display. Furthermore, for conveying score meaning, descriptive labels along the y-axis are expected to be helpful [15]. The Setting International Standards of Patient-Reported Outcomes and Quality of Life Endpoints in Cancer Clinical Trials (SISAQoL) guideline [40] reported recommendations on directionality of scale scores similar to Snyder et al. [15]. In order to enhance clinicians’ interpretation of PROMs scores they recommended to reduce the number of metrics presented (e.g. a maximum of six bars in bar graphs and 4 lines in line graphs), use coloured arrows (e.g. green for better and red for worse scores) and to accompany more complex displays like funnel plots with a detailed interpretation [40]. This guideline will contribute to standardize rules for interpretation and visualization.

Another suggestion for clarifying PROMs visualisation formats is to develop a dynamic dashboard for PROMs feedback [3, 6, 28]. This way, patients and clinicians are able to change between different graphic visualization formats. We imagine options such as comparing scores to norms or threshold scores, as not all patients may want to do so. Based on the hypothesis that serving individual preferences may facilitate interpretation accuracy, this could improve interpretation of PROMs scores as well. However, it should be taken into account that implementing a dynamic dashboard comes with challenges, like access and availability of suitable software and sufficient IT staff to support such a dashboard.

Last, the implementation of PROMs hinges on more factors than visualization of data, starting with the perceived value by patients and clinicians of discussing PROMs during clinical encounters. Nine studies included in our review noted that PROMs were perceived as valuable [4, 5, 12, 16, 25–27, 35, 36], where some studies (n = 5) showed mixed results regarding the usefulness of PROMs [1, 3, 14, 17, 19]. How patients value PROMs may determine the interest in graphic visualization of PROMs. This could have affected the presented results in this review.

This systematic review is limited by the fact that only papers published in English were included. Studies in other languages regarding locally successful implemented feedback of PROMs data might have been missed. Furthermore, in this review, no distinction is made between objective (i.e. does a person actually interpret scores correctly), and subjective (i.e. does the person says he or she interprets scores correctly) interpretation accuracy. Only two included studies made a distinction between the different types of interpretation accuracy [4, 17]; for the other studies, based on the description of the study methods, we believe objective interpretation accuracy was assessed. Therefore, future research may investigate potential differences between actual and perceived interpretation accuracy.

Another potential bias is that for the majority of included studies it remains unclear how questions about preferences and interpretation of the presented visualization format(s) were framed to the study population. Besides, study participants may have had different background knowledge about graphic visualization formats, presentation of formats, and the content of PROMs data. This could confound our findings about how data and graphs were interpret, understood, and valued.

Furthermore, this review did not consider different levels of patients’ health literacy, since this information was not presented in the majority of articles reviewed. Patients with a higher level of health literacy can have different preferences compared to patients with lower levels of health literacy [3]. Some patients with limited health literacy may not understand the longitudinal nature of data from left to right nor the temporal connection between different graphical elements [28]. Therefore, healthy literacy should be included as factor in studies dealing with data interpretation, for example measured through the use of the validated Health Literacy Questionnaire (HLQ) [41]. Furthermore, health literacy can differ among age, gender, and/or education level and therefore should be studied among these separate groups of patients in relation to PROMs visualization [42, 43].

In conclusion, there was no predominant graphical visualization format approach in terms of preferences or interpretation accuracy for both patients and clinicians. To guide the clinical interpretation of scores during clinical encounters, PROMs scores can be compared to patients’ previous scores or mean scores from a norm population, or compared to comparator scores or score thresholds. Furthermore, the use of colors, threshold lines, or circles around alarming scores can visualize the clinical meaning of PROMs scores that have been compared to previous scores, norms, thresholds, or reference populations. Finally, detailed clarification of each graph may be essential for accurate interpretation. Visualization strategies should therefore include descriptions of PROMs directionality as well as standard descriptive labels, brief definitions, and presentation of a limited number of symptoms in a graph.

## Supplementary Information

**Additional file 1: Table S1**. Search strategy for MEDLINE (accessed through PubMed), Embase (accessed through Ovid Platform), PsycINFO (accessed through Ovid Platform) and CINAHL.

## Data Availability

Data sharing is not applicable to this article as no datasets were generated or analysed during the current study.

## Abbreviations

CASP: Critical appraisal skills programme
CDAI: Clinical disease activity index
HER: Electronic Health Record
EORTC-QLQ-C30: European organization for research and treatment of cancer quality of life questionnaire Core 30
HADS: Hospital anxiety and depression scale
HCD: Human centered design
HRQoL: Health-related quality of life
HUI: Health utility index
Health-ITUES: Health IT usability evaluation scale
HLQ: Health literacy questionnaire
JHCRN: Johns Hopkins clinical research network
N/A: Not applicable
PRISMA: Preferred reporting items for systematic reviews and meta-analysis
PROMs: Patient reported outcome measurements
PROMIS: Patient-reported outcomes measurement information system
QoL: Quality of life
REALM-SF: Rapid estimate of adult literacy in medicine short form
SISAQoL: Setting international standards of patient-reported outcomes and quality of life endpoints in cancer clinical trials
SD: Standard deviation
USA: United States of America
WHOQOL: World health organization quality of life

## References

[1] Fischer KI, De Faoite D, Rose M (2020). Patient-reported outcomes feedback report for knee arthroplasty patients should present selective information in a simple design - findings of a qualitative study. J Patient Rep Outcomes.

[2] Hildon Z, Allwood D, Black N (2012). Making data more meaningful: patients' views of the format and content of quality indicators comparing health care providers. Patient Educ Couns.

[3] Izard J, Hartzler A, Avery DI, Shih C, Dalkin BL, Gore JL (2014). User-centered design of quality of life reports for clinical care of patients with prostate cancer. Surgery.

[4] Kuijpers W, Giesinger JM, Zabernigg A, Young T, Friend E, Tomaszewska IM (2016). Patients' and health professionals' understanding of and preferences for graphical presentation styles for individual-level EORTC QLQ-C30 scores. Qual Life Res.

[5] Oerlemans S, Arts LP, Horevoorts NJ, van de Poll-Franse LV (2017) "Am I normal?" The Wishes of patients with lymphoma to compare their patient-reported outcomes with those of their peers. J Med Internet Res. 19(8):e288. 10.2196/jmir.7079 PMC5575418 28811271

[6] Hartzler AL, Izard JP, Dalkin BL, Mikles SP, Gore JL (2016). Design and feasibility of integrating personalized PRO dashboards into prostate cancer care. J Am Med Inform Assoc.

[7] Snyder C, Brundage M, Rivera YM, Wu AW (2019) A PRO-cision medicine methods toolkit to address the challenges of personalizing cancer care using patient-reported outcomes: introduction to the supplement. Med Care. 57:S1-s7 10.1097/MLR.0000000000001089 PMC7400766 30985589

[8] Snyder CF, Blackford AL, Aaronson NK, Detmar SB, Carducci MA, Brundage MD (2011). Can patient-reported outcome measures identify cancer patients' most bothersome issues?. J Clin Oncol.

[9] Berry DL, Blumenstein BA, Halpenny B, Wolpin S, Fann JR, Austin-Seymour M (2011). Enhancing patient-provider communication with the electronic self-report assessment for cancer: a randomized trial. J Clin Oncol.

[10] Yang LY, Manhas DS, Howard AF, Olson RA (2018). Patient-reported outcome use in oncology: a systematic review of the impact on patient-clinician communication. Supp Care Cancer.

[11] Chen J, Ou L, Hollis SJ (2013). A systematic review of the impact of routine collection of patient reported outcome measures on patients, providers and health organisations in an oncologic setting. BMC Health Serv Res.

[12] Brundage M, Leis A, Bezjak A, Feldman-Stewart D, Degner L, Velji K (2003). Cancer patients' preferences for communicating clinical trial quality of life information: a qualitative study. Qual Life Res.

[13] Bantug ET, Coles T, Smith KC, Snyder CF, Rouette J, Brundage MD (2016). Graphical displays of patient-reported outcomes (PRO) for use in clinical practice: What makes a pro picture worth a thousand words?. Patient Educ Couns.

[14] Damman OC, Verbiest MEA, Vonk SI, Berendse HW, Bloem BR, de Bruijne MC (2019). Using PROMs during routine medical consultations: The perspectives of people with Parkinson's disease and their health professionals. Health Expect.

[15] Snyder C, Smith K, Holzner B, Rivera YM, Bantug E, Brundage M (2019). Making a picture worth a thousand numbers: recommendations for graphically displaying patient-reported outcomes data. Qual Life Res.

[16] Brundage MD, Smith KC, Little EA, Bantug ET, Snyder CF (2015). Communicating patient-reported outcome scores using graphic formats: results from a mixed-methods evaluation. Qual Life Res.

[17] Loth FL, Holzner B, Sztankay M, Bliem HR, Raoufi S, Rumpold G (2016). Cancer patients' understanding of longitudinal EORTC QLQ-C30 scores presented as bar charts. Patient Educ Couns.

[18] Smith KC, Brundage MD, Tolbert E, Little EA, Bantug ET, Snyder CF (2016). Engaging stakeholders to improve presentation of patient-reported outcomes data in clinical practice. Supp Care Cancer.

[19] van Overveld LFJ, Takes RP, Vijn TW, Braspenning JCC, de Boer JP, Brouns JJA (2017). Feedback preferences of patients, professionals and health insurers in integrated head and neck cancer care. Health Expect.

[20] Tolbert E, Brundage M, Bantug E, Blackford AL, Smith K, Snyder C (2019). In proportion: approaches for displaying patient-reported outcome research study results as percentages responding to treatment. Qual Life Res.

[21] Basch E, Barbera L, Kerrigan CL, Velikova G (2018). Implementation of patient-reported outcomes in routine medical care. Am Soc Clin Oncol Educ Book.

[22] Shamseer L, Moher D, Clarke M, Ghersi D, Liberati A, Petticrew M, et al (2015) Preferred reporting items for systematic review and meta-analysis protocols (PRISMA-P) 2015: elaboration and explanation. Bmj.350:g7647 10.1136/bmj.g7647 25555855

[23] Bramer WM, Giustini D, de Jonge GB, Holland L, Bekhuis T (2016). De-duplication of database search results for systematic reviews in EndNote. J Med Libr Assoc.

[24] Nadelson S, Nadelson LS (2014). Evidence-based practice article reviews using CASP tools: a method for teaching EBP. Worldviews Evid Based Nurs.

[25] Ragouzeos D, Gandrup J, Berrean B, Li J, Murphy M, Trupin L (2019). "Am I OK?" using human centered design to empower rheumatoid arthritis patients through patient reported outcomes. Patient Educ Couns.

[26] Geerards D, Pusic A, Hoogbergen M, van der Hulst R, Sidey-Gibbons C (2019) Computerized Quality of Life Assessment: A Randomized Experiment to Determine the Impact of Individualized Feedback on Assessment Experience. J Med Internet Res.21(7):e12212. 10.2196/12212 PMC6657452 31298217

[27] Grossman LV, Feiner SK, Mitchell EG, Masterson Creber RM (2018). Leveraging patient-reported outcomes using data visualization. Appl Clin Inform.

[28] Liu LH, Garrett SB, Li J, Ragouzeos D, Berrean B, Dohan D (2020). Patient and clinician perspectives on a patient-facing dashboard that visualizes patient reported outcomes in rheumatoid arthritis. Health Expect.

[29] Tolbert E, Brundage M, Bantug E, Blackford AL, Smith K, Snyder C (2018). Picture this: presenting longitudinal patient-reported outcome research study results to patients. Med Decis Making.

[30] Brundage M, Feldman-Stewart D, Leis A, Bezjak A, Degner L, Velji K (2005). Communicating quality of life information to cancer patients: a study of six presentation formats. J Clin Oncol.

[31] Brundage MD, Smith KC, Little EA, Bantug ET, Snyder CF (2015) Board PRODPSA. Communicating patient-reported outcome scores using graphic formats: results from a mixed-methods evaluation. Qual Life Res. 24(10):2457–72 10.1007/s11136-015-0974-y PMC4891942 26012839

[32] McNair AG, Brookes ST, Davis CR, Argyropoulos M, Blazeby JM (2010). Communicating the results of randomized clinical trials: do patients understand multidimensional patient-reported outcomes?. J Clin Oncol.

[33] Santana MJ, Feeny DH (2009). Using the health utilities index in routine clinical care: process, feasibility, and acceptability: a randomized controlled trial. Patient.

[34] Snyder CF, Smith KC, Bantug ET, Tolbert EE, Blackford AL, Brundage MD (2017). What do these scores mean? Presenting patient-reported outcomes data to patients and clinicians to improve interpretability. Cancer.

[35] Jagsi R, Chiang A, Polite BN, Medeiros BC, McNiff K, Abernethy AP (2013). Qualitative analysis of practicing oncologists' attitudes and experiences regarding collection of patient-reported outcomes. J Oncol Pract.

[36] Hartzler AL, Chaudhuri S, Fey BC, Flum DR, Lavallee D (2015). Integrating Patient-Reported Outcomes into Spine Surgical Care through Visual Dashboards: Lessons Learned from Human-Centered Design. EGEMS (Wash DC).

[37] Brundage M, Blackford A, Tolbert E, Smith K, Bantug E, Snyder C (2018). Presenting comparative study PRO results to clinicians and researchers: beyond the eye of the beholder. Qual Life Res.

[38] Brundage M, Bass B, Jolie R, Foley K (2011). A knowledge translation challenge: clinical use of quality of life data from cancer clinical trials. Qual Life Res.

[39] Snyder CF, Aaronson NK (2009). Use of patient-reported outcomes in clinical practice. Lancet.

[40] Hancock SL, Ryan OF, Marion V, Kramer S, Kelly P, Breen S, et al (2020) Feedback of patient-reported outcomes to healthcare professionals for comparing health service performance: a scoping review. BMJ Open. 10(11):e038190 10.1136/bmjopen-2020-038190 PMC7684821 33234623

[41] Osborne RH, Batterham RW, Elsworth GR, Hawkins M, Buchbinder R (2013). The grounded psychometric development and initial validation of the Health Literacy Questionnaire (HLQ). BMC Public Health.

[42] Williams MV, Davis T, Parker RM, Weiss BD (2002). The role of health literacy in patient-physician communication. Fam Med.

[43] Clouston SAP, Manganello JA, Richards M (2017). A life course approach to health literacy: the role of gender, educational attainment and lifetime cognitive capability. Age Ageing.

