# Supplementary material for: Visualization formats of patient-reported outcome measures in clinical practice: a systematic review about preferences and interpretation accuracy
Source: J Patient Rep Outcomes. 2022 Mar 3;6:18. doi: 10.1186/s41687-022-00424-3 (PMC8894516; doi:10.1186/s41687-022-00424-3)
Supplement: Supplementary file 1 — Additional file 1: Table S1. Search strategy for MEDLINE (accessed through PubMed), Embase (accessed through Ovid Platform), PsycINFO (accessed through Ovid Platform) and CINAHL. [file 41687_2022_424_MOESM1_ESM.docx]

Supplementary Table 1: Search strategy for MEDLINE (accessed through PubMed), Embase (accessed through Ovid Platform), PsycINFO (accessed through Ovid Platform) and CINAHL.

| **MEDLINE (PubMed)** | **Embase (Ovid Platform)** | **PsycINFO (Ovid platform)** | **CINAHL** |
| --- | --- | --- | --- |
| (("Patient Outcome Assessment"[Mesh] OR "Patient Reported Outcome Measures"[Mesh] OR patient reported outcome*[tiab] OR patient reported out-come*[tiab] OR patient report outcome*[tiab] OR patient report out-come*[tiab] OR PRO-CTCAE[tiab] OR patient outcome assessment*[tiab] OR patient outcomes assessment[tiab] OR health related quality of life[tiab] OR HRQOL[tiab] OR ((symptom*[tiab] OR functioning[tiab] OR self-report*[tiab]) AND (surveillance[tiab] OR supervision*[tiab] OR monitor*[tiab]))) AND ("Computer Graphics"[Mesh] OR "Data Display"[Mesh] OR graph*[tiab] OR data display*[tiab] OR information display*[tiab] OR data presentation*[tiab] OR visual*[tiab] OR personalized feedback[tiab] OR picture*[tiab]) AND ("Comprehension"[Mesh] OR "Communication"[Mesh:NoExp] OR "Health Communication"[Mesh] OR comprehension[tiab] OR understanding[tiab] OR feedback[tiab] OR interpret*[tiab] OR graph literacy[tiab] OR graphic literacy[tiab] OR health communication[tiab]) AND ((neoplasms[mesh] OR adenoma*[tiab] OR angiosarcoma*[tiab] OR astrocyomas*[tiab] OR blastoma*[tiab] OR cancer*[tiab] OR carcinom*[tiab] OR carcinosarcoma*[tiab] OR chordoma*[tiab] OR chondrosarcoma*[tiab] OR dysgerminoma[tiab] OR ependymoma*[tiab] OR essential thrombocythaemia[tiab] OR germinoma*[tiab] OR GIST[tiab] OR GISTs[tiab] OR glioma*[tiab] OR glomangiosarcoma*[tiab] OR haemangioblastoma*[tiab] OR hemangiosarcoma*[tiab] OR hepatoblastoma*[tiab] OR hodgkin disease[tiab] OR hodgkin's disease[tiab] OR hodgkins disease[tiab] OR leiomyosarcoma*[tiab] OR leukemi*[tiab] OR lymphangiosarcoma*[tiab] OR lymphom*[tiab] OR malignan*[tiab] OR medulloblastoma*[tiab] OR melanom*[tiab] OR meningioma*[tiab] OR mesenchymoma*[tiab] OR mesonephroma*[tiab] OR mesothelioma*[tiab] OR metasta*[tiab] OR myelodysplastic syndrome*[tiab] OR myelofibrosis[tiab] OR myeloma*[tiab] OR neoplas*[tiab] OR nephroblastoma*[tiab] OR neuroma*[tiab] OR nsclc[tiab] OR oligodendroglioma*[tiab] OR oncolog*[tiab] OR osteosarcoma*[tiab] OR plasmacytoma*[tiab] OR polycythaemia vera[tiab] OR pseudomyxoma peritonei[tiab] OR rhabdomyosarcoma*[tiab] OR sarcoma*[tiab] OR teratocarcinoma*[tiab] OR teratoma*[tiab] OR tumor[tiab] OR tumors[tiab] OR tumour*[tiab]) OR ("Decision Making"[Mesh:NoExp] OR "Clinical Decision-Making"[Mesh] OR "Decision Support Techniques"[Mesh] OR decision making[tiab] OR decision support[tiab] OR clinical practice*[tiab] OR medical practice*[tiab] OR clinical use[tiab] OR clinical setting[tiab] OR care practice*[tiab]))) | ((exp Patient-Reported Outcome/ OR (patient reported outcome* OR patient reported out-come* OR patient report outcome* OR patient report out-come* OR "PRO-CTCAE" OR patient outcome assessment* OR "patient outcomes assessment" OR "health related quality of life" OR HRQOL).tw. OR ((symptom* OR functioning OR self-report*).tw. AND (surveillance OR supervision* OR monitor*).tw.)) AND (exp computer graphics/ OR exp data visualization/ OR (graph* OR data display* OR information display* OR data presentation* OR visual* OR "personalized feedback" OR picture* ).tw.) AND (exp comprehension/ OR interpersonal communication/ OR (comprehension OR understanding OR feedback OR interpret* OR "graph literacy" OR "graphic literacy" OR "health communication").tw.) AND ((exp neoplasm/ OR (adenoma* OR angiosarcoma* OR astrocyomas* OR blastoma* OR cancer* OR carcinom* OR carcinosarcoma* OR chordoma* OR chondrosarcoma* OR dysgerminoma OR ependymoma* OR essential thrombocythaemia OR germinoma* OR GIST OR GISTs OR glioma* OR glomangiosarcoma* OR haemangioblastoma* OR hemangiosarcoma* OR hepatoblastoma* OR hodgkin disease OR hodgkin's disease OR hodgkins disease OR leiomyosarcoma* OR leukemi* OR lymphangiosarcoma* OR lymphom* OR malignan* OR medulloblastoma* OR melanom* OR meningioma* OR mesenchymoma* OR mesonephroma* OR mesothelioma* OR metasta* OR myelodysplastic syndrome* OR myelofibrosis OR myeloma* OR neoplas* OR nephroblastoma* OR neuroma* OR nsclc OR oligodendroglioma* OR oncolog* OR osteosarcoma* OR plasmacytoma* OR polycythaemia vera OR pseudomyxoma peritonei OR rhabdomyosarcoma* OR sarcoma* OR teratocarcinoma* OR teratoma* OR tumor OR tumors OR tumour*).tw.) OR (decision making/ OR exp medical decision making/ OR exp clinical decision support system/ OR ("decision making" OR "decision support" OR clinical practice* OR medical practice* OR "clinical use" OR "clinical setting" OR care practice*).tw.))) | ((exp Patient Reported Outcome Measures/ OR exp health related quality of life/ OR (patient reported outcome* OR patient reported out-come* OR patient report outcome* OR patient report out-come* OR "PRO-CTCAE" OR patient outcome assessment* OR "patient outcomes assessment" OR "health related quality of life" OR HRQOL).tw. OR ((symptom* OR functioning OR self-report*).tw. AND (surveillance OR supervision* OR monitor*).tw.)) AND (exp graphical displays/ OR exp visual displays/ OR (graph* OR data display* OR information display* OR data presentation* OR visual* OR "personalized feedback" OR picture* ).tw.) AND (exp comprehension/ OR communication/ OR (comprehension OR understanding OR feedback OR interpret* OR "graph literacy" OR "graphic literacy" OR "health communication").tw.) AND ((exp neoplasms/ OR (adenoma* OR angiosarcoma* OR astrocyomas* OR blastoma* OR cancer* OR carcinom* OR carcinosarcoma* OR chordoma* OR chondrosarcoma* OR dysgerminoma OR ependymoma* OR essential thrombocythaemia OR germinoma* OR GIST OR GISTs OR glioma* OR glomangiosarcoma* OR haemangioblastoma* OR hemangiosarcoma* OR hepatoblastoma* OR hodgkin disease OR hodgkin's disease OR hodgkins disease OR leiomyosarcoma* OR leukemi* OR lymphangiosarcoma* OR lymphom* OR malignan* OR medulloblastoma* OR melanom* OR meningioma* OR mesenchymoma* OR mesonephroma* OR mesothelioma* OR metasta* OR myelodysplastic syndrome* OR myelofibrosis OR myeloma* OR neoplas* OR nephroblastoma* OR neuroma* OR nsclc OR oligodendroglioma* OR oncolog* OR osteosarcoma* OR plasmacytoma* OR polycythaemia vera OR pseudomyxoma peritonei OR rhabdomyosarcoma* OR sarcoma* OR teratocarcinoma* OR teratoma* OR tumor OR tumors OR tumour*).tw.) OR (exp decision making/ OR exp decision support systems/ OR ("decision making" OR "decision support" OR clinical practice* OR medical practice* OR "clinical use" OR "clinical setting" OR care practice*).tw.))) | (TI(patient reported outcome* OR patient reported out-come* OR patient report outcome* OR patient report out-come* OR "PRO-CTCAE" OR patient outcome assessment* OR "patient outcomes assessment" OR "health related quality of life" OR HRQOL) OR AB(patient reported outcome* OR patient reported out-come* OR patient report outcome* OR patient report out-come* OR "PRO-CTCAE" OR patient outcome assessment* OR "patient outcomes assessment" OR "health related quality of life" OR HRQOL OR ((TI(symptom* OR functioning OR self-report*) OR AB(symptom* OR functioning OR self-report*)) AND (TI(surveillance OR supervision* OR monitor*) OR AB(surveillance OR supervision* OR monitor*))))) AND (TI(graph* OR data display* OR information display* OR data presentation* OR visual* OR "personalized feedback" OR picture*) OR AB(graph* OR data display* OR information display* OR data presentation* OR visual* OR "personalized feedback" OR picture*)) AND (TI(comprehension OR understanding OR feedback OR interpret* OR "graph literacy" OR "graphic literacy" OR "health communication") OR AB(comprehension OR understanding OR feedback OR interpret* OR "graph literacy" OR "graphic literacy" OR "health communication")) AND ((TI(adenoma* OR angiosarcoma* OR astrocyomas* OR blastoma* OR cancer* OR carcinom* OR carcinosarcoma* OR chordoma* OR chondrosarcoma* OR dysgerminoma OR ependymoma* OR essential thrombocythaemia OR germinoma* OR GIST OR GISTs OR glioma* OR glomangiosarcoma* OR haemangioblastoma* OR hemangiosarcoma* OR hepatoblastoma* OR hodgkin disease OR hodgkin's disease OR hodgkins disease OR leiomyosarcoma* OR leukemi* OR lymphangiosarcoma* OR lymphom* OR malignan* OR medulloblastoma* OR melanom* OR meningioma* OR mesenchymoma* OR mesonephroma* OR mesothelioma* OR metasta* OR myelodysplastic syndrome* OR myelofibrosis OR myeloma* OR neoplas* OR nephroblastoma* OR neuroma* OR nsclc OR oligodendroglioma* OR oncolog* OR osteosarcoma* OR plasmacytoma* OR polycythaemia vera OR pseudomyxoma peritonei OR rhabdomyosarcoma* OR sarcoma* OR teratocarcinoma* OR teratoma* OR tumor OR tumors OR tumour*) OR AB(adenoma* OR angiosarcoma* OR astrocyomas* OR blastoma* OR cancer* OR carcinom* OR carcinosarcoma* OR chordoma* OR chondrosarcoma* OR dysgerminoma OR ependymoma* OR essential thrombocythaemia OR germinoma* OR GIST OR GISTs OR glioma* OR glomangiosarcoma* OR haemangioblastoma* OR hemangiosarcoma* OR hepatoblastoma* OR hodgkin disease OR hodgkin's disease OR hodgkins disease OR leiomyosarcoma* OR leukemi* OR lymphangiosarcoma* OR lymphom* OR malignan* OR medulloblastoma* OR melanom* OR meningioma* OR mesenchymoma* OR mesonephroma* OR mesothelioma* OR metasta* OR myelodysplastic syndrome* OR myelofibrosis OR myeloma* OR neoplas* OR nephroblastoma* OR neuroma* OR nsclc OR oligodendroglioma* OR oncolog* OR osteosarcoma* OR plasmacytoma* OR polycythaemia vera OR pseudomyxoma peritonei OR rhabdomyosarcoma* OR sarcoma* OR teratocarcinoma* OR teratoma* OR tumor OR tumors OR tumour*)) OR (TI("decision making" OR "decision support" OR clinical practice* OR medical practice* OR "clinical use" OR "clinical setting" OR care practice*) OR AB("decision making" OR "decision support" OR clinical practice* OR medical practice* OR "clinical use" OR "clinical setting" OR care practice*))) |

Supplementary Table 2: Full description of study characteristics of the included studies.

| **Author + year** | **Primary study goal/ research questions** | **Study population** | **Study design** | **Setting*** | **Outcome measures** | **General conclusion** |  |
| --- | --- | --- | --- | --- | --- | --- | --- |
| Brundage, 2003 (12) | To explore patients' attitudes toward, and preferences for, 10 visual and written formats for communicating Health Related Quality of Life (HRQoL) information. | N=14 men and N=19 women with variety of cancer diagnoses, post treatment ≥6 months earlier, Canada. | Qualitative study | - Clinical care  - Six months after treatment | - Themes relating the presented information  - Perceived usefulness | Simple formats (graphs or written text) were generally preferred to more complex graphical information, regardless of educational level. Patients did not wish to receive HRQoL information out of context or without explanation. |  |
| Brundage, 2005 (31) | To determine which formats for presenting HRQoL data are interpreted most accurately and are most preferred by patients. | Patients with variety of cancer diagnosis, previously treated (N=198), Canada. | Structured interview | - Clinical care  - After treatment | - Interpretations accuracy  - Ease-of-use  - Helpfulness | Format, age, and education were independent predictors for interpretation accuracy. Patients prefer a simple linear representation of group mean HRQoL scores, and 98% accurately interpret data presented in this format, irrespective of age and educational level. |  |
| Brundage, 2015 (16) | To investigate the interpretability of current PRO data presentation formats. | N=50 patients with variety of cancer diagnoses; N=20 clinicians in active practice, JHCRN**. | Cross-sectional mixed-methods study | - Clinical care  - Six months after treatment | - Interpretation accuracy  - Understanding  - Usefulness  - Helpful  - Confusing format attributes | Patients and clinicians prefer line graphs across group-level data and individual-level data formats, but clinicians prefer greater detail (e.g., statistical details) for group-level data. |  |
| Brundage, 2018 (37) | To evaluate the interpretation accuracy and perceived clarity of various strategies for displaying clinical trial PRO findings. | Oncology clinicians (N=233) and PRO researchers (N=248), JHCRN**. | Electronic survey; interviews | Clinical care | - Interpretation accuracy  - Clarity ratings | Graphic formats for presenting PRO data differ in how accurately they are interpreted and how clear they are. |  |
| Damman, 2019 (14) | To investigate:  (a) How patients and clinicians think about using PROMs during consultations;  (b) For which purpose patients and clinicians use PROMs during consultations;  (c) How patients interpret PROMs information presented in various formats. | Interviews: patients with Parkinson's disease (N=13) and clinicians (N=14).  Survey: patients (N=115), the Netherlands. | Semi‐structured interviews; survey | Clinical care | Key themes:  - Correct interpretation  - Perceived usefulness  - Attitude  - Preferences  - Decisions | When used in routine medical consultations, PROMs have potential to support shared decision-making and facilitate patient‐clinician communication.  Training seems needed for both patients and clinicians for actual discussion and proper interpretation. |  |
| Fischer, 2020 (1) | To develop a PRO feedback report for mobile devices that is comprehensible and provides valuable information for patients after knee arthroplasty. | Orthopedic patients (N=8), Germany. | Iterative development process (literature review and think-aloud interview) | Clinical care | - Value  - Report preferences  - Comprehensibility  - Degree of informative content | A PRO feedback report for patients for mobile app use should take into account the heterogeneous user group (demographics such as age and experience with mobile devices). Information should be presented in a simple way to be comprehensible and of value to patients. |  |
| Geerards, 2019 (27) | To assess the impact of tailored multimodal feedback and computerized adapted testing (CAT) on user experience in HRQoL assessment using validated PROMs. | N=1386 participants from the general population, United Kingdom (UK). | RCT | Hypothetical setting | - Perceived acceptability  - Engagement  - Clarity  - Accuracy | Using tailored text-based feedback to contextualize numeric scores maximized the acceptability of electronic HRQoL assessment. Improving user experience may increase response rates and reduce attrition in research and clinical use of PROMs. CAT administration was associated with a modest decrease in assessment length but did not improve user experience. |  |
| Grossman, 2018 (28) | To identify the design requirements for an interface that assists patients with PRO survey completion and interpretation; to build and evaluate the interface of PROMs feedback. | Interview: N=13 patients with heart failure and N=11 clinicians, study location or country was not described.  Usability testing: N=12 patients with heart failure, USA. | Human-centered design (HCD) (interviews and design-testing) | - Clinical care  - Confirmed diagnosis | - Value and challenges of PRO use  - Comprehension  - Usability (usability, credibility, loyalty, appearance, and overall quality)  - Visualization preferences | Aiding comprehension is a design requirement (e.g. by incorporating visualizations into our interface design). Future interfaces may benefit from employing strategies such as visualization to aid comprehension and engage patients with surveys. |  |
| Hartzler, 2015 (36) | To share lessons learned from engaging clinicians to inform design of visual dashboards. | Clinicians: N=12 for interviews, N=40 for surveys and consensus meeting, N=9 for user testing, study location or country was not described. | HCD (interviews, surveys, consensus meeting, user testing) | Clinical care | - User interface design features and preferences  - Considerations use of PROs | Engaging clinicians as stakeholders is a critical step toward the design of user-friendly HIT that is accepted, usable, and has the potential to enhance quality of care and patient outcomes. |  |
| Hartzler, 2016 (6) | To conduct a HCD to engage patients, providers, and interaction design experts in the development of visual “PRO dashboards” that illustrate personalized trends in patients’ HRQoL following prostate cancer treatment. | Focus groups (N=60 patients).  N=50 prostate cancer patients and N=50 clinicians, study location or country was not described. | HCD (interviews, surveys, consensus meeting, user testing) | - Clinical care  - During treatment | - Helpfulness  - Preferences  - Recommendations for clinical use | Our approach illustrates stakeholder engagement methods, innovative dashboard prototypes, and design insights on meaningful dashboard content, format, tailoring, and clinical use. These contributions establish guidance for optimizing the design of PRO displays and, support patient-centered care. |  |
| Hildon, 2012 (2) | To explore patients’ views of different formats and content of data displays of PROMs. | N=45 patients undergone or planning knee surgery in six focus groups, UK. | Qualitative study | Hypothetical setting | Key themes:  - Un/familiarity  - Explanation  - Less is more  - Dis/enabling cognitive processing  - Inferred meaning - Popular resonance | Aspects of familiarity, meaningfulness (whether and how displays resonated with participants), and cognitive or choice processes informed views of displays. |  |
| Izard, 2014 (3) | To develop graphic dashboards of questionnaire responses from patients with prostate cancer to facilitate clinical integration of HRQoL measurement. | N=50 prostate cancer patients and N=50 providers from Seattle, USA. | Qualitative study (interviews, surveys, focus groups) | - Clinical care  - During or after treatment | - Comprehension  - Preferences | A dynamic HRQoL dashboard that permits a base patient-centered report in bar charts that can be toggled to other formats and include error bars that frame comparison group scores. |  |
| Jagsi, 2012 (35) | To investigate practicing oncologists view on incorporating routine collection of PROs into cancer care. | N=17 oncologists, USA. | Qualitative study (semi-structured interviews) | Clinical care | Key themes:  - Knowledge of PRO’s  - Understanding of PRO’s  - Experience with PROs  - Value/doubts of PROs  - PRO implementation | Oncologists had variable understanding of details of PROs but, when introduced to the concept, recognized utility in improving efficiency and thoroughness of patient encounters if implemented properly. |  |
| Kuijpers, 2016 (4) | To investigate patients’ and clinicians’ understanding of and preferences for different graphical presentation styles for individual-level EORTC QLQC30 scores. | N=548 cancer patients in four European countries and N=227 clinicians, the Netherlands. | Observational study (survey) | - Clinical care  - Hypothetical setting  - During or after treatment | - Objective and self-rated understanding  - Preferences | Discrepancy between participants’ high self-rated and relatively low objective understanding of graphical presentation of PRO results highlights the need to provide sufficient guidance when presenting results. It may be appropriate to adapt presentation of PRO results to individual preferences. |  |
| Liu et al., 2020 (29) | To develop Rheumatoid Arthritis (RA) ‘dashboard’ that could facilitate conversations about PROs and is acceptable to a wide range of patients, including English and Spanish speakers, with adequate or limited health literacy. | N=25 RA patients and N=11 clinicians from two academic rheumatology clinics, California. | HCD (focus groups) | Hypothetical setting | - Comprehension  - Preferences  - Desired uses | Ability to customize data display preferences is important in tailoring the dashboard to patients with diverse needs and preferences. Special attention to feasibility concerns voiced by clinicians (dashboard work as intended, anticipated difficulties in importing accurate data from the EHR and incorporating it effectively into workflows). |  |
| Loth, 2016 (17) | To investigate patients’ understanding of graphical presentations of longitudinal EORTC QLQ-C30 scores. | N=40 brain tumor patients, Austria. | Semi-structured interviews | - Clinical care  - After treatment | - Understanding  - Self-rating/ objectively  - Opinions on congruency between scores and self-rated health | Patients are able to understand their HRQoL results when presented graphically and are able to interpret important changes. |  |
| McNair, 2010 (32) | To assess patients’ understanding of multidimensional PROs in a graphical format. | Patients with esophageal and gastric cancer (N=132), UK. | Semi-structured interviews | - Clinical care  - Hypothetical setting  - During or after treatment | Key themes:  - Understanding  - Interpretation | Most patients understand graphical multidimensional PROs, although a smaller majority were able to interpret more complex, or simultaneous, presentations. |  |
| Oerlemans, 2017 (5) | To investigate whether patients with lymphoma wished to receive PRO feedback, including the option to compare their scores with those of their peers, and how this feedback was evaluated. | Lymphoma patients (N=64), the Netherlands. | Observational study (survey) | - Clinical care  - During or after treatment | - HRQoL scores  - Feedback preferences (what reference population, for which symptoms) | A high number of patients wished to receive PRO feedback. Patients reported the comparison of their scores versus a lymphoma reference cohort as most valuable. |  |
| Ragouzeos, 2019 (26) | To develop a “dashboard” for RA patients to display relevant PRO measures for discussion during a routine RA clinical visit. | Patients with rheumatology (N=45) and providers (N=12), USA. | HCD (interviews, focus groups, prototype testing) | - Clinical care  - During treatment | - Informational needs and preferences | RA patients and providers shared the goals of assessing wellbeing and developing a personalized treatment plan. Conflicting views of which data were most important for decision-making and for answering the patient’s overarching question of “Am I OK?” |  |
| Santana, 2009 (33) | To describe the process, feasibility and acceptability of use of the Health Utilities Index (HUI) in routine clinical care. | Pre- and post-heart and -lung transplant patients (N=151), Canada. | RCT | - Clinical care  - Pre and post lung and heart transplantation | - Process, feasibility and acceptability of HUI use | Patients quickly learned how to complete HUI questionnaires on the computer. Clinicians found that the information provided was valuable and have subsequently incorporated the use of the HUI in clinical care. |  |
| Smith, 2016 (18) | To improve formats for presenting individual-level PRO data (for patient monitoring) and group-level PRO data (for reporting comparative clinical studies). | N=40 clinicians in active practice and N=39 patients diagnosed with cancer ≥6 months previously, not currently receiving chemotherapy/radiation or within 6 months of surgery, JHCRN**. | Workgroups; Semi-structured, one-on-one interviews | - Clinical care  - Hypothetical setting | Key themes:  - Interpretation issues  (score meaning, directional inconsistency)  - Highlighting and interpreting score differences | Variations in interpretation accuracy demonstrate the importance of presenting PRO data in ways that promote understanding and use. |  |
| Snyder, 2017 (34) | To test approaches for presenting PRO data to improve interpretability. | N=627 cancer patients/survivors, N=236 oncology clinicians, and N=250 PRO researchers for survey.  N=10 patients and N=10 clinicians for interviews, JHCRN**. | Mixed-methods study (Internet survey, one-to-one interviews) | - Clinical care  - During or after treatment | - Interpretation accuracy  - Clarity  - Most useful format | Higher=better directionality was both more accurately interpreted and more likely to be rated clear. Threshold lines are more likely to be rated very clear than red circles or green shading. Threshold lines were selected as “most useful” across respondent types and directionality. |  |
| Tolbert, 2018 (30) | To identify the association of PRO score directionality and score norming on a) how accurately PRO scores are interpreted and b) how clearly they are rated by patients, clinicians, and PRO researchers. | N=629 patients (various oncologic diagnoses), N=139 oncology clinicians, and N=249 PRO researchers, JHCRN**. | Cross-sectional, mixed-methods study (survey; One-on-one cognitive interviews) | - Clinical care  - Hypothetical setting  - During or after treatment | - Interpretation accuracy  - Clarity | For communicating PROs as line graphs in patient educational materials and decision aids, these results support using graphs with higher scores consistently indicating better outcomes. |  |
| Tolbert, 2019 (20) | To identify best practices for presenting PRO results expressed as proportions of patients with changes from baseline (improved/ stable/ worsened) for use in patient educational materials and decision aids. | N=629 patients (various oncologic diagnoses, treated), N=139 oncology clinicians, and N=249 PRO researchers, JHCRN**. | Cross-sectional, mixed-methods study (Survey; One-on-one cognitive interviews) | - Clinical care  - Hypothetical setting  - During or after treatment | - Interpretation accuracy  - Clarity  - Format preference | For communicating PROs as proportions changed in patient educational materials and decision aids, these results support the use of pie charts. |  |
| van Overveld, 2017 (19) | To investigate the preferences of receiving feedback between stakeholders. | N=37 patients, medical specialists, allied health professionals and health insurers in the Netherlands | Semi- structured interviews | - Clinical care  - After treatment | Key themes:  - Preferences to receive feedback and how  - Aspect to prefer feedback on | Feedback preferences differ between stakeholders. Tailored reports are recommended. Effects of audit and feedback can be improved by adapting feedback format and contents to preferences of stakeholders. |  |

*Setting: This measure describes whether the study is a hypothetical setting and/or regular clinical care setting and at which stage of disease or treatment the study was performed.

** JHCRN – Johns Hopkins Clinical Research Network: A consortium of academic and community health systems in the US mid-Atlantic with clinics outside the USA as well.

Supplementary Table 3: Critical Appraisal Skills Programme (CASP) quality assessment according to the study design performed, for all included studies (n=25).

| **Author** | **Study design** | **Quality scoring criteria, according to the study design*** | | | | | | | | | | | | |
| --- | --- | --- | --- | --- | --- | --- | --- | --- | --- | --- | --- | --- | --- | --- |
|  |  | **1** | **2** | **3** | **4** | **5** | **6** | **7** | | **8** | **9** | **10** | **11** | **12** |
| Brundage, 2003 (12) | Qualitative | Y | Y | Y | Y | Y | Y | ? | | Y | Y | V | n/a | n/a |
| Brundage, 2005 (31) | Qualitative | Y | Y | Y | Y | Y | ? | Y | | Y | Y | V | n/a | n/a |
| Brundage, 2015 (16) | Qualitative | Y | Y | Y | Y | Y | Y | Y | | Y | Y | V | n/a | n/a |
| Brundage, 2018 (37) | Qualitative | Y | Y | Y | Y | Y | ? | Y | | Y | Y | V | n/a | n/a |
| Damman, 2019(14) | Qualitative | Y | Y | Y | Y | Y | Y | Y | | Y | Y | V | n/a | n/a |
| Fischer, 2020 (1) | Qualitative | Y | Y | Y | Y | Y | ? | Y | | Y | Y | V | n/a | n/a |
| Geerards, 2019 (27) | RCT | Y | Y | Y | Y | Y | Y | No difference was found in perceived accuracy of the graphical feedback scores of the WHOQOL-CAT and WHOQOL-BREF. 82.4% thought the graphical feedback was accurate, and 92.9% thought the graphical feedback was clear. | | Mean CAT feedback accuracy 2.9, SD 0.9; mean fixed feedback accuracy 3.1, SD 1.0; P=.05; Δ=0.06, Δ 95% CI 0.00-0.12 | N | Y | Y | n/a |
| Grossman, 2018 (28) | Qualitative | Y | Y | Y | Y | Y | ? | Y | | Y | Y | V | n/a | n/a |
| Hartzler, 2015 (36) | Qualitative | Y | Y | Y | Y | Y | ? | Y | | N | Y | V | n/a | n/a |
| Hartzler, 2016 (6) | Qualitative | Y | Y | Y | Y | Y | ? | Y | | Y | Y | V | n/a | n/a |
| Hildon, 2012 (2) | Qualitative | Y | Y | Y | Y | Y | ? | Y | | N | Y | V | n/a | n/a |
| Izard, 2014 (3) | Qualitative | Y | Y | Y | Y | Y | ? | Y | | Y | Y | V | n/a | n/a |
| Jagsi, 2012 (35) | Qualitative | Y | Y | Y | Y | Y | ? | N | | Y | Y | V | n/a | n/a |
| Kuijpers, 2016 (4) | RCT | Y | Y | ? | ? | Y | Y | For those receiving non-colored charts, almost half of the patients did not have a preference (x2 = 30.5, p<0.001). For patients who received colored charts, bar charts were favored (x2 = 49.2, p<0.001). Functioning scales were better understood than symptom scales (absolute scores t = 3.08, p = 0.002; overall change t = 4.91, p<0.001; specific change t = 9.93, p<0.001 | | S | Y | Y | Y | n/a |
| Liu, 2020 (29) | Qualitative | Y | Y | Y | Y | Y | ? | Y | | Y | Y | V | n/a | n/a |
| Loth, 2016 (17) | Qualitative | Y | Y | Y | Y | Y | ? | Y | | Y | Y | V | n/a | n/a |
| McNair, 2010 (32) | Qualitative | Y | Y | Y | Y | Y | ? | Y | | Y | Y | V | n/a | n/a |
| Oerlemans, 2017 (5) | Cohort | Y | Y | Y | Y | Y | Y | 80% of patients with lymphoma wished to receive PRO feedback. Patients reported the comparison of their scores versus a lymphoma reference cohort as most valuable. | | S | Y | Y | ? | Patients can monitor their symptoms at any specific point in time and are provided with information that they can use to actively engage with their physician. |
| Ragouzeos, 2019 (26) | Qualitative | Y | Y | Y | Y | Y | ? | Y | | Y | Y | V | n/a | n/a |
| Santana, 2009 (33) | RCT | Y | Y | Y | n/a | Y | Y | 89.4% of patients were happy to use a touch-screen computer. 91.4% of patients confirmed that completing the questionnaire was easy. 6.6% of patients were unwilling to complete the questionnaires. | | S | Y | Y | Y | n/a |
| Smith, 2016 (18) | Qualitative | Y | Y | Y | Y | Y | ? | Y | | Y | N | V | n/a | n/a |
| Snyder, 2017 (34) | Qualitative | Y | Y | Y | Y | Y | ? | Y | | Y | Y | V | n/a | n/a |
| Tolbert, 2018 (30) | Qualitative | Y | Y | Y | Y | Y | ? | Y | | Y | Y | V | n/a | n/a |
| Tolbert, 2019 (20) | Qualitative | Y | Y | Y | Y | Y | ? | Y | | Y | Y | V | n/a | n/a |
| Van Overveld, 2017 (19) | Qualitative | Y | Y | Y | Y | Y | ? | Y | | Y | Y | V | n/a | n/a |
| **Key:** ? = unable to tell; N = no; n/a = not applicable; P = Precise; S = sufficient; V = Valuable; Y = yes | | | | | | | | | | | | | | |
| **Abbreviations:** CI: confidence interval; RCT: randomized controlled trial | | | | | | | | | | | | | | |
| *** Critical Appraisal Skills Programme criteria per study type:** | | | | | | | | |  | | | | | |
| **Qualitative study**   1. Was there a clear statement of the aims of the research? 2. Is a qualitative/ quantitative methodology appropriate? 3. Was the research design appropriate to address the aims of the search? 4. Was the recruitment strategy appropriate to the aims of the research? 5. Was the data collected in a way that addressed the research issue? 6. Was the relationship between researcher and participants adequately considered? 7. Have ethical issues been taken into consideration? 8. Was the data analysis sufficiently rigorous? 9. Is there a clear statement of findings? 10. How valuable is the research? | | | | | | | | | **RCT**   1. Did the trial address a clearly focused issue? 2. Was the assignment of patients to treatments randomised? 3. Were all of the patients who entered the trial properly accounted for at its conclusion? 4. Were patients, health workers and study personnel ‘blind’ to treatment? 5. Were the groups similar at the start of the trial? 6. Aside from the experimental intervention, were the groups treated equally? 7. How large was the treatment effect? 8. How precise was the estimate of the treatment effect? 9. Can the results be applied to the local population, or in your context? 10. Were all clinically important outcomes considered? 11. Are the benefits worth the harms and costs? | | | | | |
| **Cohort**   1. Did the study address a clearly focused issue? 2. Was the cohort recruited in an acceptable way? 3. Was the exposure accurately measured to minimise bias? 4. Was the outcome accurately measured to minimise bias? 5. A. Have the authors identified all important confounding factors?  B. Have they take account of the confounding factors in the design and/or analysis? 6. A. Was the follow-up of subjects complete enough?  B. Was the follow-up of subjects long enough? 7. What are the results of this study? 8. How precise are the results? 9. Do you believe the results? 10. Can the results be applied to the local population? 11. Do the results of this study fit with other available evidence? 12. What are the implications of this study for practice? | | | | | | | | |  | | | | | |
